# High PKD2 predicts poor prognosis in lung adenocarcinoma via promoting Epithelial–mesenchymal Transition

**DOI:** 10.1038/s41598-018-37285-0

**Published:** 2019-02-04

**Authors:** Zhaofei Pang, Yu Wang, Nan Ding, Xiaowei Chen, Yufan Yang, Guanghui Wang, Qi Liu, Jiajun Du

**Affiliations:** 1 Institute of Oncology, Shandong Provincial Hospital Affiliated to Shandong University, Jinan, People’s Republic of China; 2 Department of Thoracic Surgery, Shandong Provincial Hospital Affiliated to Shandong University, Jinan, People’s Republic of China

## Abstract

Protein kinase D2 (PKD2) has been reported to be related with progression and invasion in various cancers. However, its prognostic value and the underlying mechanism in lung cancer remains unclear. Herein we evaluated the expression of PKD2 in lung adenocarcinoma and investigated its relationship with EMT. GSEA, TCGA and K-M plotter database were applied and revealed that high PKD2 expression predicted poor outcome and related with lymph nodes metastasis in lung cancer. IHC and qRT-PCR were performed and found PKD2 was elevated in lung adenocarcinoma and negatively related with OS (p = 0.015), PFS (p = 0.006) and the level of E-cadherin (p = 0.021). Experiment in lung adenocarcinoma cell lines demonstrated up-regulation of PKD2 led to high expression of mesenchymal markers (N-cadherin, vim, mmp9 *et al*.) and EMT transcription factors(zeb1, twist, snail), and the results were reversed when PKD2 was knocked down. Further investigation showed that abrogation of PKD2 inhibited A549 cell migration, invasion, proliferation and induced cell arrest in G2/M phase. We concluded that high expression of PKD2 was associated with poor prognosis and cancer progression in lung adenocarcinoma patients by promoting EMT.

## Introduction

Lung cancer is the most common malignancy with high mortality worldwide^1,2^. Non-small cell lung cancer (NSCLC), which is less destructive but more prevalent than small cell lung cancer, accounts for more than 80% of lung cancer. The common treatment for early stage lung cancer is surgical resection and advanced ones require chemotherapy, with or without radiotherapy^3^. Despite development of innovative therapies, the clinical outcome remains dismal, with 5-year overall survival rate less than 18%^4^. Late disease presentation, recurrence, extensive invasion and metastasis are the key causes of poor prognosis. Moreover, the development of drug resistance has emerged to be the biggest obstacle against successful chemotherapy and targeted therapy in clinical application^5^.

Epithelial–mesenchymal transition (EMT), involved in embryonic development, tissue repair, fibrosis, carcinoma progression, is considered to be an evolutionarily conserved process by which epithelial cells lose their polarity, change their morphology, and transition to migratory mesenchymal cells^6^. During EMT, cells can reorganize their cytoskeleton and undergo distinct biochemical changes that allow transition to mesenchymal cells capable to invade, resist apoptosis, and disseminate^7^. Previous studies have demonstrated that EMT to be a necessary and a key process in metastasis of cancer including NSCLC^8–10^. Furthermore, in 2015, Kari R. Fischer *et al*. reported that EMT is not required for lung metastasis but contributes to chemoresistance^11^. Cancer cell undergoing EMT may acquire altered traits, including migration, invasion, apoptotic tolerance and increased expression of chemoresistance-related genes. Therefore, investigation of the molecular mechanism underlying EMT may be of great value to provide new insights into lung cancer development as well as therapeutic strategy.

Protein kinase D (PKD), containing two cysteine-rich zinc finger domains, a pleckstrin homology domain, and a catalytic domain, is recognized as a member of serine/threonine kinase family. It can be activated by some agents such as diacylglycerol (DAG), phorbol esters, growth factors and G-protein activators through combining with N-terminal cysteine-rich motifs^12,13^, thus implicated in cancer occurrence and development by regulating cell proliferation, survival, apoptosis, migration and angiogenesis pathways^14,15^. Up to now, three members of PKD family have been identified: PKD1/PKCμ^16,17^, PKD2^18^, and PKD3/PKCν^19^. PKD1 was discovered earlier and extensively studied. In 2014, we found that PKD1 was downregulated in non-small cell lung cancer and mediated the mTORC1-S6K1 pathway in response to phorbol ester^20^. Since 2001, when the full-length cDNA of PKD2 was isolated^18^, studies have been conducted and revealed PKD2 to be high expressed in multiple tumors, including prostate, pancreatic, colorectal, gastric, hepatocellular carcinoma, malignant human lymphomas, and Glioblastoma multiforme^21–24^. PKD2 was found to be capable to orchestrate cancer cell proliferation, survival, angiogenesis, motility and immune response by regulating multiple signaling pathways (e.g., MEK/ERK, NF-κB), as well as integrating extracellular signals (e.g., signals from hypoxia, growth factors)^25,26^.

These years, studies about PKDs and EMT have been reported, and it is proved that PKDs have different effects on this transition process^27^. Indirect evidence indicated PKD2 and PKD3 were potential positive regulators of EMT^28^, whereas previous studies reported that PKD1 tended to block EMT by phosphorylation of snail and inhibiting its ability to repress the expression level of E-cadherin in cancers^29,30^. Here, we performed this study in order to evaluate the underlying roles and mechanisms of PKD2 in tumorigenesis of lung adenocarcinoma.

## Materials and Methods

### Bioinformatics analysis

To elucidate the influence of PRKD2 gene expression on the NSCLC expression profile, an enrichment analysis was conducted in the biological process of gene ontology using GSEA 3.0 tool. Data used for GSEA were accessible from The Cancer Genome Atlas (TCGA, https://cancergenome.nih.gov/) which included public genomic data. Receiver operating characteristic (ROC) curve analysis was applied to obtain appropriate cut-off value between PKD2 high and low expression groups. K-M survival curve was drawn from Kaplan-Meier Plotter database with the gene symbol “PRKD2”. We conducted the relationship between PKD2 mRNA expression level and lymph nodes metastasis by TCGA database (lung adenocarcinoma, nature 2014). A total of 513 patients were divided into two groups according to nodes metastasis or not. Adjusted p-value < 0.05 was considered to be statistically significant.

### Patients selection

Tissue samples of patients with primary lung adenocarcinoma who received operation in Shandong Provincial Hospital affiliated to Shandong University from Jan.2006 to Dec.2011 were enrolled in this study, of which 109 were for immunohistochemistry staining and 27 for evaluation of PKD2 expression by quantitative Reverse Transcription Polymerase Chain Reaction (qRT-PCR). Clinicopathological features such as age, gender, pathological stage and differential degree were collected. Staging was classified according to the eighth edition of TNM staging system of AJCC (American Joint Committee on Cancer). Overall survival (OS) referred to the time from date of surgery to death for any cause. If the patient was alive or out of touch, the endpoint of OS was the date of last follow-up. Progression-free survival (PFS) was calculated from the date of surgery to tumor progression. If there was no progression, the endpoint was the date of death or last follow-up. Informed consent was obtained from all individual participants included in the study. All procedures were conformed to the ethical guidelines of the 1975 Declaration of Helsinki and in accordance with the ethical standards of the Ethics Committee of Shandong Provincial Hospital Affiliated to Shandong University. All experimental protocols were approved by the Ethics Committee of Shandong Provincial Hospital Affiliated to Shandong University.

### Immunohistochemistry (IHC) staining

Four-μm thick tissue sections which were fixed with formalin and embedded in paraffin blocks were used for IHC staining. Anti-PKD2 antibody (dilution 1:200, Santa Cruz, CA, USA) and anti-E-cadherin antibody (dilution 1:200, Cell Signaling Technology, USA) were used as primary antibody respectively. Sections were dewaxed and received antigen repairing (high pressure method for 3 min in saline sodium citrate), whereafter they were incubated with 3% H_2_O_2_ for 30 min for quenching endogenous peroxidase activity. Subsequently, 7% goat serum was used to block cross-reactivity at 37 °C for 30 min. Biotin labelling antibody, SABC (Strept Avidin-Biotin Complex), DAB (Diaminobenzidine) were added to the samples after they were given primary antibodies and incubated at 4 °C overnight. Steps of the control group were same as above except for a substitution of primary antibody with phosphate-buffered saline (PBS).

Two observers (Pang and Du) selected five high-power fields (×400) randomly and scored these specimens according to the intensity of dyed color and the percentage of positive cells stained independently. The intensity of staining was graded as: 0, no color; 1, light yellow; 2, light brown; 3, deep brown, and grades according to positive cell numbers were determined as: 0 (<5%), 1 (5~25%), 2 (26~50%), 3 (51~75%), 4 (>75%). A range of −~+++ was determined by the final score which was calculated by multiplying the two scores: 0–1 score (−), 2 score (+), 3–4 score (++), >5 score (+++). Score 0~2 were defined as low expression, and score ≥3 were recognized as high expression.

### Cell culture, transfections

All cell lines were purchased from the American Type Culture Collection (ATCC, USA) and cultured in corresponding medium with 10% fetal bovine serum (HyClone, Logan, UT, USA) in a humidified atmosphere of 5% CO2 and 37 °C according to protocol. PKD2 plasmids (from Peter Storz, Department of Cancer Biology, Mayo Clinic) were transfected into cells using jetPRIME (Polyplus-transfection, Illkirch, France) for transient transfection according to the manufacturer’s instructions. Further analyses were performed 48 h after transfection.

Lentiviruses were produced using HEK293T cells to knock down PKD2. Briefly 6 × 10^6^ cells were plated in 10 cm plates and simultaneously transfected with 5 μg PKD2 shRNA plasmid (from Derek C. Radisky, Department of Cancer Biology, Mayo Clinic), 5 μg of pSPAX2 (Addgene, USA) and 2 ug pMD2.G (Addgene, USA). EZ Trans (Life iLAB Biotech, Shanghai, China) was used for transfection according to manufacturer’s instructions. Medium was changed after 24 hours and lentivirus containing medium was collected 48 hours post transfection. For lentiviral transduction in a 6-well plate, 600 μl regular medium, 400 μl viral supernatant and 6 μg/ml polybrene were used.

### RNA extraction, cDNA synthesis and real-time qRT-PCR analysis

Total RNA was extracted with RNAiso Plus kit (Takara, Tokyo, Japan) and mRNA was reverse-transcribed using PrimeScript™ RT reagent Kit with gDNA Eraser (Takara) according to the manufacturer’s protocol. Gene expression levels were assayed by qRT-PCR using the Roche LightCycler ® 480 system and SYBR Premix Ex Taq^TM^ (Takara).

The primers used for quantitative PCR are listed as followed:

PRKD2–1: 5′>GTCATTGACAAACTGCGCTTCC<3′

PRKD2-2: 5′>GCGTCTCGAACATGCACTCC<3′

CDH2-1: 5′>CGGTTTCATTTGAGGGCACA<3′

CDH2-2: 5′>TTGGAGCCTGAGACACGATT<3′

Vimentin-1: 5′>TGCAGGCTCAGATTCAGGAA<3′

Vimentin-2: 5′>CTCCGGTACTCAGTGGACTC<3′

Snail1-1: 5′>CCCCAATCGGAAGCCTAACT<3′

Snail1-2: 5′>GACAGAGTCCCAGATGAGCA<3′

Snail2-1: 5′>CCTGGTTGCTTCAAGGACAC<3′

Snail2-2: 5′>AGCAGCCAGATTCCTCATGT<3′

MMP9-1: 5′>TGCCACTTCCCCTTCATCTT<3′

MMP9-2: 5′>CGTCCTGGGTGTAGAGTCTC<3′

MMP3-1: 5′>CCTGGAAATGTTTTGGCCCA<3′

MMP3-2: 5′>TCATCTTGAGACAGGCGGAA<3′

Zeb1-1: 5′>CAGGGAGGAGCAGTGAAAGA<3′

Zeb1-2: 5′>CTCTTCAGGTGCCTCAGGAA<3′

NF-κB-1: 5′>CAGCTACGCCTTCTCGGTCT<3′

NF-κB-2: 5′>ACTGTCCATTTTCTCCTTCTCTGG<3′

Twist-1: 5′>CAGCTACGCCTTCTCGGTCT<3′

Twist-2: 5′>ACTGTCCATTTTCTCCTTCTCTGG<3′

18S rRNA-1: 5′>AAACGGCTACCACATCCAAG<3′

18S rRNA-2: 5′>CCTCCAATGGATCCTCGTTA<3′

All assays were performed in triplicate and analysis was performed using Microsoft Excel and the 2^−ΔΔCt^ method to obtain relative quantitation (RQ) values, with 18S rRNA used as endogenous control.

### Western blot analysis

For activation of PKD2 phosphorylation, cells at 70–80% confluence were maintained in serum-free medium for 6 hours and subsequently stimulated with Phorbol 12-myristate 13-acetate (PMA, Sigma, USA). Protein samples were isolated by the RIPA lysis buffer and the concentrations were measured by BCA protein assay according to the manufacturer’s protocol. Total protein extract were separated on 10% SDS-PAGE gels and subsequently transferred to nitrocellulose (NC) membrane (Millipore, Billerica, USA). After blocking in 5%BSA solution, the membrane was incubated with first antibody at 4 °C overnight. The primary antibodies were listed as followed: PKD2 and N-cadherin (Santa Cruz, CA, USA: 1:500); E-cadherin and vimentin (Cell Signaling Technology, USA: 1:1000); GAPDH, NF-κB, and Twist (Santa Cruz, CA, USA: 1:1000); p-PKD2 (Abcam, Cambridge, UK: 1:1000). After washing with TBST, the membrane was incubated with corresponding HRP-labeled secondary antibody (Santa Cruz, CA, USA: 1:10000) for 1 h. The detection was performed with ECL kit and FluorChem E system (Proteinsimple, CA, USA).

### Cell migration and invasion assays

Cell migration and invasion assays were analyzed using the Transwell chambers assay (Costar, Corning Inc., Corning, NY, USA), with or without coated Matrigel (Corning). A549 Cells were plated at a density of 40 k per well(invasion assay) and 5 k per well(migration assay) in the upper chamber in 0.1% BSA F12K medium without serum. The lower chamber of the transwell was then added with 500 complete F12K medium with 10% FBS. After 18 h the noninvading cells were remove from the top well with a cotton swab. The bottom cells were fixed with methanol at −20 °C for 30 min, stained with 0.1% crystal violet at room temperature for 1 hour and photographed. The total invaded cells per chamber were counted and the data presented represents triplicated ±SEM for each experimental condition.

### Proliferation assays and cell cycle

Cells were resuspended and seeded in 96-well plate with a density of 1000 cells per well. After the cells adherence, they were fixed with 10% trichloroacetic acid for at least 24 h, after which they were stained by Sulforhodamine B sodium salt (SRB) (Sigma, USA) for 30 min and washed three times by 1% (vol/vol) acetic acid. About 24 hours later after the plates were dry, 150 ul 10 mmol/L Tris were added to each well and the absorbance was measured at 510 nm^31^.

For EDU staining, cells were seeded at 50–70% confluence and then stained with EDU at 37 °C for 2 hours. After fixed with 4% paraformaldehyde 15 min at room temperature, they were permeabilized with 0.3% triton X-100. The cells were subsequently added with click reaction buffer following with Hoechst staining for 10 min according to protocol (Beyotime, Shanghai, China).

Cell cycle analysis was performed with PI/RNase Staining Buffer (Becton, Dickinson, Franklin Lakes, NJ, USA) and detected by MUSE Cell Analyzer (Merck Millipore, Darmstadt, Germany) according to the manufacturer’s instructions. All experiment were tripled and the present data were presented mean ± SEM.

### Statistical analysis

Chi-square test or Fisher’s exact test were employed to investigate the relationship between PKD2 and other clinicopathological variables, and the prognostic value of PKD2 in lung adenocarcinomas were analyzed by Kaplan-Meier (K-M) survival curve. Hazard ratio (HR) with its 95% confidence interval (CI) for describing association of variables and survival were calculated by univariate and multivariate cox regression methods. The significance between different groups were calculated by t-test. All statistical calculations were performed by SPSS (version 20.0) software (Inc., Chicago, IL, USA), Microsoft Excel and GraphPad Prism 7.0 (GraphPad Software, Inc., La Jolla, CA, USA). p < 0.05 was considered to be statistically significant.

## Results

### Expression and Prognostic value of PKD2 in lung cancer

**Figure 1:**
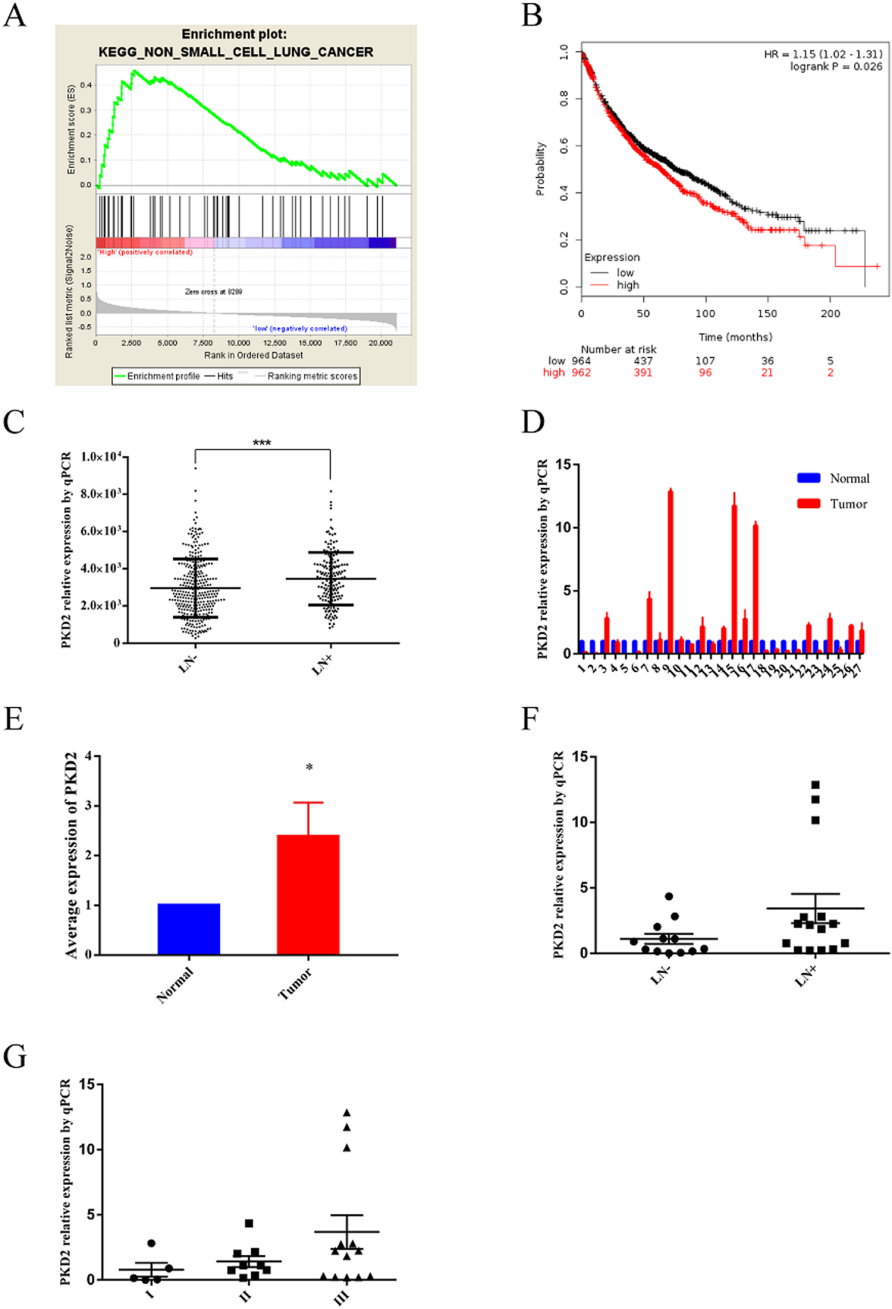
Expression and prognostic role of PKD2 in lung cancer. **(A)** GSEA enrichment analysis of co-expression genes. **(B)** Survival curve from Kaplan Meier-plotter database, HR = 1.15 (1.02–1.31), p = 0.026. **(C)** Relative PKD2 mRNA expression between groups with lymph nodes metastasis group or not (TCGA, nature 2014). **(D**–**G)** Expression of PKD2 in lung adenocarcinoma tissues and its distribution in different lymph nodes metastasis and pathological stage groups.

To explore oncogenic signaling related to PKD2, we performed GSEA and found that NSCLC related genes were mainly enriched in tumors with high level of PKD2 (Fig. 1A). The survival curve from Kaplan-Meier Plotter database demonstrated that high level of PKD2 predicted poor outcome in lung cancer patients (Fig. 1B). The data extracted from TCGA showed that high expression of PKD2 was significantly related with lymph nodes metastasis with p < 0.001 (Fig. 1C). Twenty-seven pair of tumors matched with adjacent nontumor tissues were included to investigate the expression of PKD2 in lung adenocarcinoma (Table 1). As shown in Fig. 1D,E, the mRNA level of PKD2 was significantly elevated in lung adenocarcinoma tissues compared with adjacent tissues (p < 0.05). Furthermore, high expression of PKD2 insignificantly indicated lymph nodes metastasis and advanced stage (Fig. 1F,G).

**Table 1:** The characteristics of patients for qRT-PCR and IHC.

Clinicopathologic features	IHC	qRT-PCR
	Number (%)	Number (%)
All patients	109	27
**Age, year**		
>60	47 (43.1%)	18 (66.7%)
≤60	62 (56.9%)	9 (33.3%)
**Gender**		
Male	54 (49.5%)	11 (40.7%)
Female	55 (50.5%)	16 (59.3%)
**Differential degree**		
High	23 (21.1%)	2 (7.4%)
Moderate	61 (56.0%)	13 (48.1%)
Low	25 (22.9%)	12 (44.4%)
**T stage**		
T1-2	93(85.32%)	21 (77.8%)
T3-4	16 (14.7%)	6 (22.2%)
**N status**		
N−	58 (53.2%)	12 (44.4%)
N+	51 (46.8%)	15 (55.6%)
**Pathological stage**		
I	47 (43.1%)	5 (18.5%)
II	22 (20.2%)	9 (33.3%)
III	40 (36.7%)	13 (48.1%)

### Expression of PKD2 and E-cadherin by IHC

**Figure 2:**
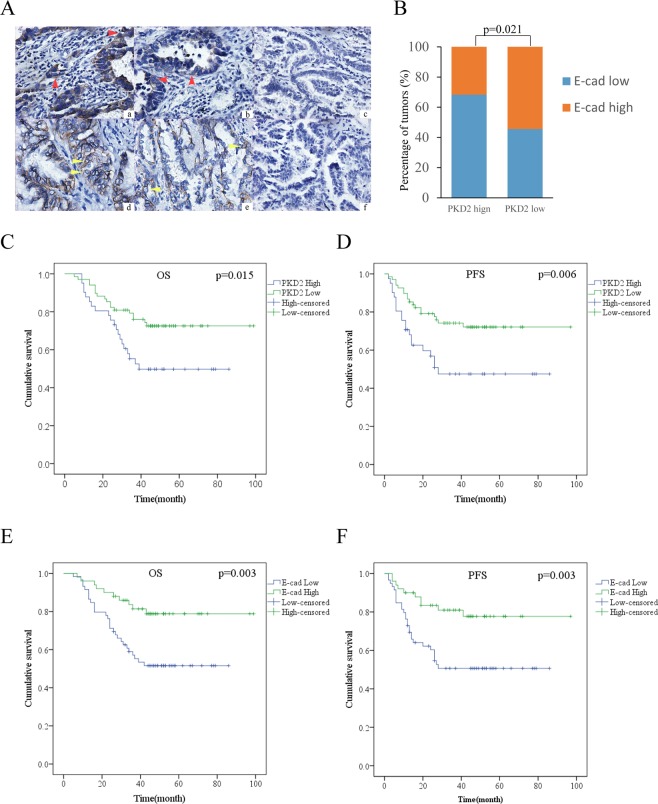
The prognostic value of PKD2 and its relationship with E-cadherin by IHC. (**A**) Representative images of different expressions of PKD2 and E-cadherin. a. high expression of PKD2 (400×); b low expression of PKD2 (400×); c. negative expression of PKD2 for control group (400×); d high expression of E-cadherin (400×); e low expression of E-cadherin (400×); f negative expression of E-cadherin for control group (400×). The red arrows indicated PDK2-expressing cells while the yellow arrows indicated E cadherin-expressing cells. (**B**) PKD2 was negatively related with E-cadherin (p = 0.021). (**C,D**) Kaplan-Meier curves for PKD2 expression and OS, PFS. High expression of PKD2 was associated with shorter OS (p = 0.015) and PFS (p = 0.006). (**E,F**) Kaplan-Meier curves for E-cadherin expression and OS, PFS. High expression of E-cadherin was related to positive prognosis of patients with lung adenocarcinoma (OS: p = 0.003; PFS: p = 0.003).

The characteristics of patients IHC were listed in Table 1. PKD2 staining was observed in the cytoplasm of cancer cells, while E-cadherin staining was observed in a membranous pattern. Representative IHC staining images of PKD2 and E-cadherin were shown in Fig. 2A.

In our study, 41 (37.6%) out of 109 samples showed high expression of PKD2, and E-cadherin was highly expressed in 50 (45.9%) specimens. Twenty eight (68.3%) samples of PKD2 high expression group were found to be E-cadherin low expressed, while 13 (31.7%) had high expression of E-cadherin. In the low expression of PKD2 group, high expression of E-cadherin was found in 37 (54.4%) sections, with the other 31 (45.6%) specimens displaying low expression (Fig. 2B). The result demonstrated that PKD2 was negatively associated with E-cadherin (p = 0.021).

### Survival analysis of PKD2, E-cadherin expression in lung adenocarcinoma

Kaplan-Meier survival curves demonstrated high level of PKD2 expression to be a significantly unfavorable prognostic factor for overall survival (OS) (p = 0.015) and progression free survival (PFS) (p = 0.006), while high expression of E-cadherin predicted good outcomes in patients with lung adenocarcinoma (OS: p = 0.003; PFS: p = 0.003) (Fig. 2C–F).

Results obtained by univariate cox regression analysis shown in Table 2 indicated that OS and PFS were correlated with PKD2 expression (OS: HR = 2.150, 95% CI: 1.136–4.071, p = 0.019; PFS: HR = 2.382, 95% CI: 1.257–4.516, p = 0.008), E-cadherin expression (OS: HR = 0.357, 95% CI: 0.173–0.736, p = 0.005; PFS: HR = 0.355, 95% CI: 0.172–0.731, p = 0.005). No significant correlations were found between survival and gender, age, or smoking history. Further multivariate analysis using a Cox regression model demonstrated that high expression of E-cadherin, III stage of pathological TMN stage, poor differential degree were independent prognostic factors for survival (Table 2).

**Table 2:** Univariate and multivariate analysis of prognostic factors for overall survival (OS) and progression free survival (PFS) in lung.

Characteristics	OS				PFS			
	Univariate		Multivariate		Univariate		Multivariate	
	HR (95% CI)	p	HR (95% CI)	p	HR (95% CI)	p	HR (95% CI)	p
**Age, year**								
≤60	1		1		1		1	
>60	1.153 (0.608–2.186)	0.662	*	*	1.243 (0.685–2.348)	0.503	*	*
**Gender**								
Female	1		1		1		1	
Male	1.258 (0.665–2.379)	0.48	*	*	1.234 (0.652–2.332)	0.518	*	*
**Smoking history**								
No	1		1				1	
Yes	1.554 (0.821–2.939)	0.176	*	*	1.523 (0.806–2.881)	0.195	*	*
**PKD2 expression**								
Low	1		1		1		1	
High	2.150 (1.136–4.071)	0.019	1.344 (0.67–2.696)	0.405	2.382 (1.257–4.516)	0.008	1.577 (0.757–3.285)	0.224
**Pathological TMN stage**								
I	1		1		1		1	
II	2.369 (0.913–6.148)	0.076	1.815 (0.685–4.807)	0.231	2.436 (0.938–6.330)	0.068	1.717 (0.629–4.684)	0.194
III	3.946 (1.799–8.655)	0.001	2.554 (1.103–5.912)	0.029	3.673 (1.674–8.058)	0.001	2.325 (1.005–5.379)	0.083
**Differential degree**								
Well	1		1		1		1	
Moderate	9.492 (1.279–70.440)	0.028	6.653 (0.876–50.522)	0.067	9.776 (1.317–72.570)	0.026	7.204 (0.948–54.768)	0.056
Poor	22.252 (2.929–169.029)	0.003	13.904 (1.763–109.638)	0.012	21.075 (2.776–160.005)	0.003	13.685 (1.741–107.553)	0.013
**E-cadherin expression**								
Low	1		1		1		1	
High	0.357 (0.173–0.736)	0.005	0.370 (0.174–0.786)	0.010	0.355 (0.172–0.731)	0.005	0.426 (0.199–0.914)	0.028

Abbreviation: PKD2-protein kinase d2; OS-overall survival; PFS-progression free survival; HR-hazard ratio; CI-confidence interval.

### The expression of PKD2 in lung cancer cell lines and its activation by PMA

The expression of total PKD2 was assessed in different cell lines at protein and mRNA level as shown in Supplementary Fig. S1A,B. Qin Hao *et al*. reported that A549 human lung carcinoma cells expressed an abundant amount of PKD2^32^, which was consistent with our result that PKD2 was highly expressed in A549 cells in both protein and mRNA level. A549 and another lung adenocarcinoma cell line PC9 were selected for further investigation. Previous studies have shown that treatment of cells with phorbol esters induces a dramatic activation of PKD2^33^. We also determined the effect of PMA on the phosphorylation of PKD2 at C-terminal Ser^876^. Serum starved A549 and PC9 cells were treated with PMA at increasing concentrations (0, 5, 10, 50, 100 nM) for 30 min and then harvested. The activation of p-PKD2 was found to be concentration-dependent manner with maximal effect at a concentration of PMA 50 nM. Meanwhile, we examined the PKD2 phosphorylation in response to different treating time of 50 nM PMA. PMA stimulated PKD2 phosphorylation increased dramatically to maximum level at 60 min for A549 and 10 min for PC9 (Supplementary Fig. S1C–J).

### PKD2 contributes to EMT

**Figure 3:**
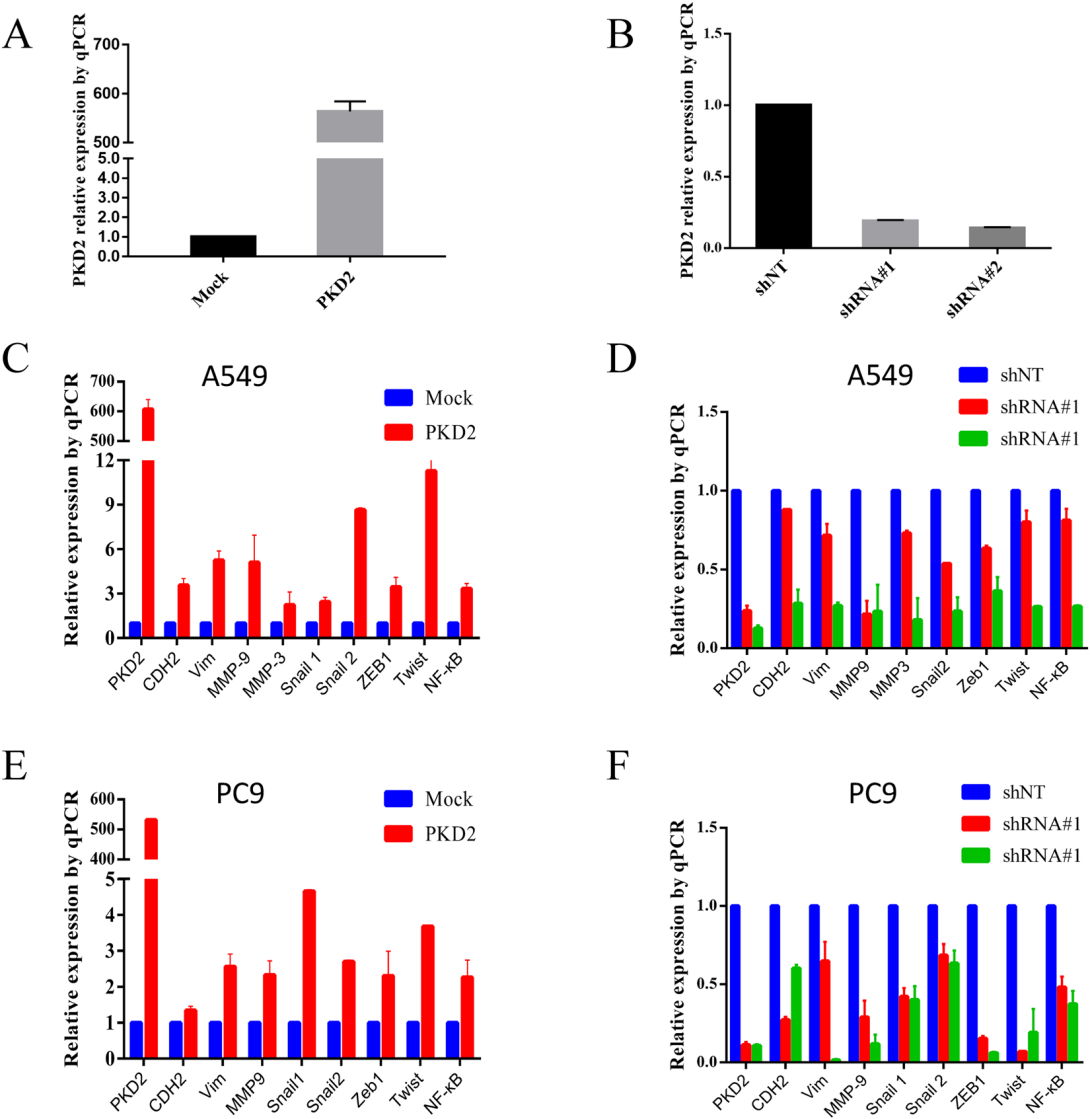
The expression of PKD2 and EMT markers after PKD2 knock-in and knock-down by qRT-PCR. **(A,B)** Evaluation of efficiency of PKD2 knock-in and Knock down in A549 cells. **(C,E)** The expression of EMT markers and transcription factors were significantly increased when PKD2 was upregulated. **(D,F)** The expression of EMT markers and transcription factors were significantly decreased when PKD2 was knocked down.

**Figure 4:**
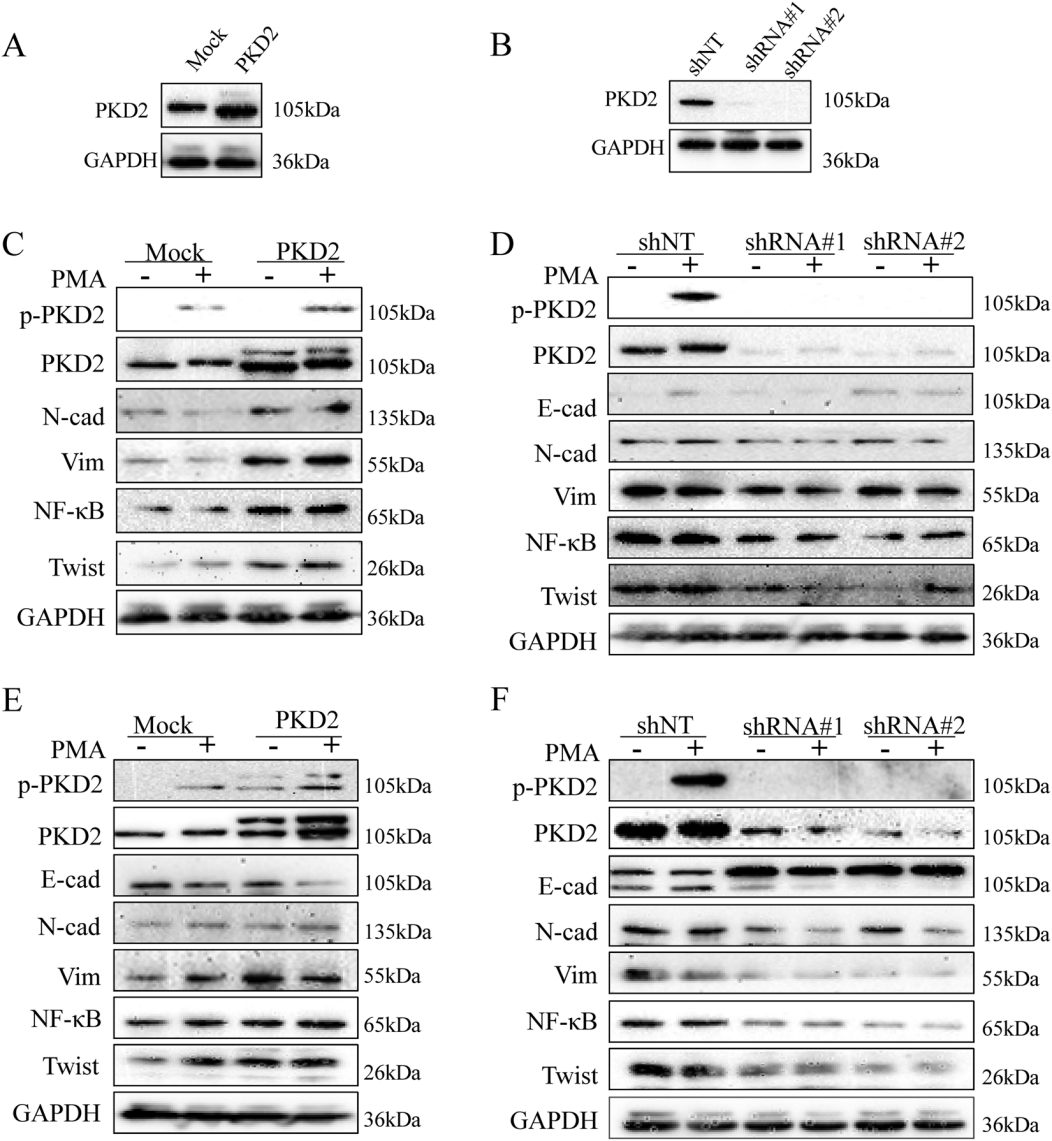
The expression of PKD2 and EMT markers after PKD2 knock-in and knock-down by western blot. **(A,B)** Evaluation of efficiency of PKD2 knock-in and Knock down in A549 cells. **(C,E)** The expression of EMT markers and transcription factors were significantly increased when PKD2 was upregulated. **(D,F)** The expression of EMT markers and transcription factors were significantly decreased when PKD2 was knocked down.

The role of PKD2 in promoting EMT in lung adenocarcinoma cell lines was next explored. We first examined the knock-in and knock-down efficiency in A549 cell. As shown in Figs 3 and 4, PKD2 was overexpressed after transient transfection and remarkably knocked down after infection of lentivirus both at mRNA and protein level. Furthermore, qPCR and western blot were employed to assess the changes of EMT transcription factor (TFs) and EMT markers. Consequently, the mesenchymal markers (e.g. N-cadherin, vim.) and EMT TFs (e.g. twist, snail.) were significantly positively correlated with the expression of PKD2 in both A549 and PC9 cells, which indicated that PKD2 promoted EMT in lung adenocarcinoma. As previous studies have demonstrated NF-κB downstream effector of PKD2 in prostate cancer cells^26^, it was also included in our experiment. Coincidentally, the change of NF-κB was in consistent with PKD2, which might provide evidence that PKD2 could promote EMT through NF-κB pathway.

### PKD2 positively regulated migration, invasion and proliferation in lung adenocarcinoma cells

**Figure 5:**
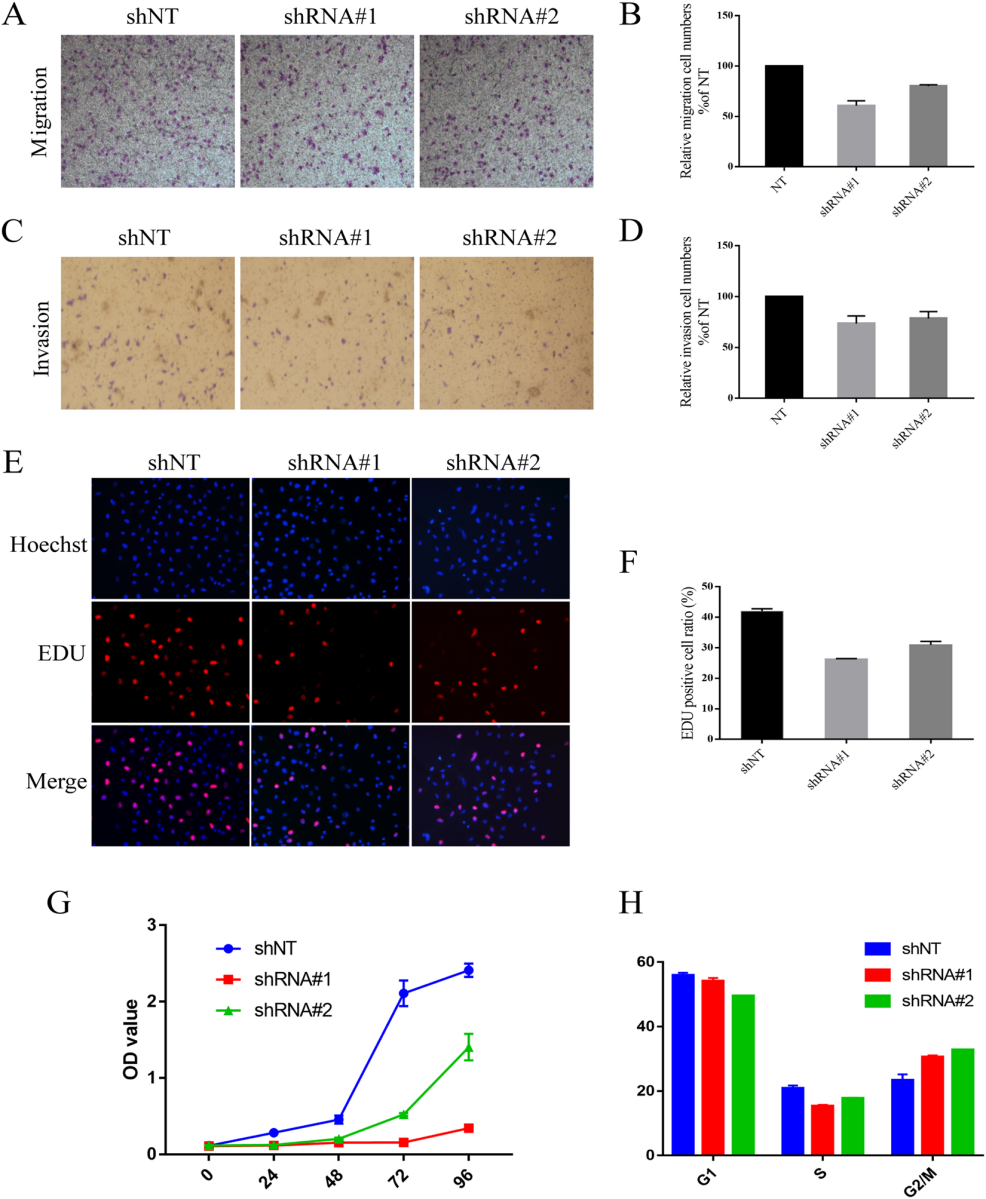
Abrogation of PKD2 inhibit migration, invasion and proliferation in A549 cells. (**A**–**D**) The levels of cell migration and invasion in A549 cells were analyzed using the Transwell chambers assay with or without coated Matrigel. **(E**–**G)** Lower PKD2 contributed to less proliferation ability of A549 detected by EDU and SRB staining. **(H)** Abrogation of PKD2 induced A549 arrest in G2/M phase.

The migration and invasion ability of A549 cell were tested by Transwell assay with or without coated Matrigel. As shown in Fig. 5A–D, A549 cells exhibited the lower ability for migration and invasion after knock down of PKD2 compared with the control cells. EDU and SRB staining were applied to evaluate the proliferation ability of A549 cells in response to expression change of PKD2. We found that lower PKD2 could suppress the duplication of cells (Fig. 5E–G). To further investigate the underlying mechanism, the cell cycle assay was conducted and revealed that PKD2 abrogation induced the arrest of A549 cells in G2/M phase (Fig. 5H).

## Discussion

Since Sabine Sturany and his colleagues isolated the full-length cDNA of a novel human protein kinase gene and named it PKD2 due to the strongest homology to the serine threonine protein kinases PKD/PKCμ and PKCν in 2001, different isoforms of PKD family was found expressed in a tissue-specific manner and could also have distinct functions^18^. Studies have been conducted to investigate the effect of PKD2 in various cancers. In 2006, overexpression of PKD2 was reported to be associated with proliferation and invasion of BON endocrine cells (established from a human pancreatic carcinoid tumor)^34^. Kovalevska L.M. *et al*. found that expression levels of PKD2 were different in different malignant human lymphomas^23^. Later in 2012, Zhipeng Zou published an article and demonstrated that PKD2 and PKD3 coordinated to promote prostate cancer cell invasion through NF-κB and HDAC1-mediated expression and activation of uPA^21^. Subsequently PKD2 was revealed to be related to migration and invasion of glioblastoma cells *in vitro* in 2013^24^. And recently, overexpression of PKD2 was reported in hepatocellular carcinoma (HCC) and was proposed associated with metastasis in HCC^22^. However, there were no studies investigating the correlation between PKD2 expression and prognosis in cancer patients directly. And the role of PKD2 in lung cancer remained unclear. In the present study, we explored the prognostic value and potential mechanisms in lung adenocarcinomas *in vitro*.

Data from Kaplan-Meier Plotter database and TCGA suggested that high expression of PKD2 might predict poor prognosis and indicate lymph nodes metastasis in lung cancer. Then we collected 27 pair of lung adenocarcinoma tissues for qPCR and 109 tumor samples to perform immunohistochemistry staining, which revealed that PKD2 was high expressed in lung adenocarcinoma and predicted negative outcome for these patients.

However, the mechanism of how PKD2 expression affected prognosis of lung adenocarcinoma patients was still unknown. Previous studies reported that PKD2 was implicated in cell proliferation, apoptosis, migration, angiogenesis and EMT^22,35–37^. In S Borges’s study, MDA-MB-231 cells which did not express PKD1 treated with the pan-PKD inhibitor CRT0066101 showed a change in morphology (increased spreading of cells) that was indicative for a decrease in motility and EMT compared to control cells treated with DMSO^28^. Recently, Yun Zhu *et al*. demonstrated for the first time that PKD2 regulated EMT and invasiveness of HCC and the expression of PKD2 was related to the metastasis and recurrence potential of HCC. Their findings identified a previously unrecognized mechanism for PKD2 regulating EMT. Enhanced by TNF-α, PKD2 bound directly to p110α and p85 subunits of PI3K promoting PI3K/Akt/GSK-3β signaling pathway and contributed to EMT and invasiveness of HCC^22^. Thus we also studied expression level of E-cadherin by IHC to identify the relationship between PKD2 and EMT. Results showed high expression of E-cadherin was significantly associated with extensive OS and PFS, while PKD2 expression had significantly negative correlation with expression level of E-cadherin. In order to verify the effect of PKD2 in EMT, we also conducted PCR and western blot in lung adenocarcinoma cell lines. Results indicated that up-regulation of PKD2 lead to high expression of mesenchymal markers and EMT transcription factors, while reversed results obtained when PKD2 knocked down. Moreover, our study indicated NF-κB might be the underlying signal pathway, by which PKD2 regulated EMT. Further investigation demonstrated that abrogation of PKD2 inhibited A549 cell migration, invasion and proliferation. While Ninel Azoitei *et al*. reported PKD2 siRNA lead to an accumulation of glioblastoma cells in G1 phase by a down-regulation of cyclin D1 expression^38^, we found that lower PKD2 induced A549 cells arrest in G2/M phase, which was consistent with the reports that PKD2 modulated cell cycle by stabilizing Aurora A kinase at centrosomes^39^.

So we surmised that PKD2 was a positive regulator of EMT, through which high expression of PKD2 contributed to poor prognosis of patients with lung adenocarcinoma. While various signaling pathways such as TGFs, BMPs, FGF, EGF, HGF, Wnt/beta-catenin and Notch were involved in the process of EMT^40,41^, deep mechanism should be explored further.

## Supplementary information

Supplementary info file

## Data Availability

Data from TCGA database is available in http://cancergenome.nih.gov/. Data from Kaplan-Meier Plotter database is available in http://www.kmplot.com/analysis/.
