# Supplementary material for: High PKD2 predicts poor prognosis in lung adenocarcinoma via promoting Epithelial–mesenchymal Transition
Source: Sci Rep. 2019 Feb 4;9:1324. doi: 10.1038/s41598-018-37285-0 (PMC6362154; doi:10.1038/s41598-018-37285-0)
Supplement: Supplementary file 1 — Supplementary info file [file 41598_2018_37285_MOESM1_ESM.pdf]

Supplementary info file

High PKD2 predicts poor prognosis in lung adenocarcinoma via promoting Epithelial–mesenchymal Transition

Zhaofei Pang1,*, Yu Wang1,*, Nan Ding1, Xiaowei Chen1, Yufan Yang1, Guanghui Wang2, Qi Liu1, Jiajun Du1,2, §, MD, PHD

1 Institute of Oncology, Shandong Provincial Hospital Affiliated to Shandong University, Jinan, People's Republic of China;
2 Department of Thoracic Surgery, Shandong Provincial Hospital Affiliated to Shandong University, Jinan, People's Republic of China；
* These authors contributed equally to this work.
§Corresponding authors:
Jiajun Du, 324 Jingwu Road, Jinan, 250021, People's Republic of China, Phone: +86-0531-6877-7100; E-mail: dujiajun568@163.com

Supplementary figure 1

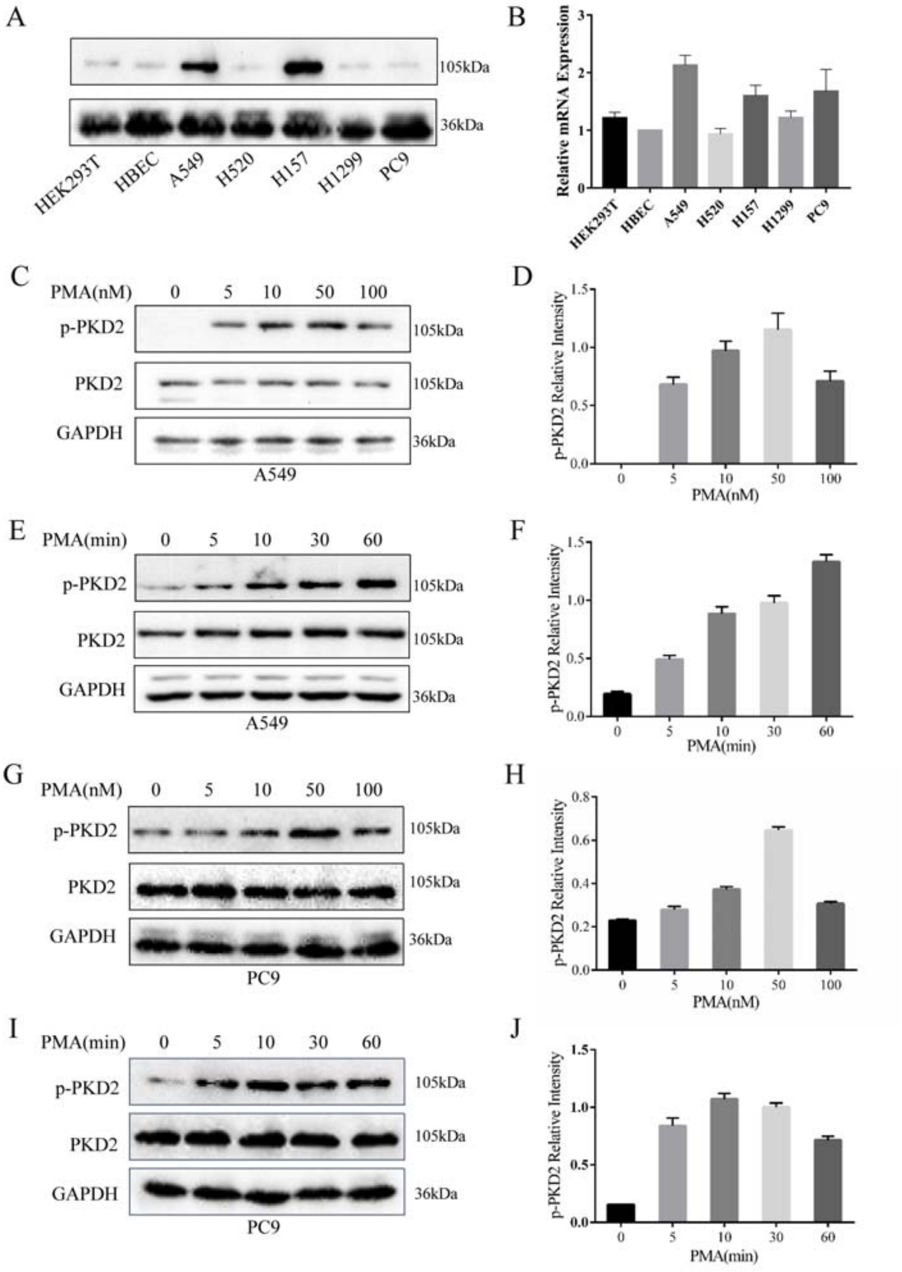

Supplementary Figure 1: Expression and phosphorylation of PKD2 in lung cancer cell lines. (A and B) The expression levels of PKD2 were detected by western blot and qRT–PCR in human lung cancer cells. (C and G) Serum-starved A549 and PC9 cells were stimulated by 0, 5, 10, 50, 100nmol/L PMA for 30min to detect p-PKD2 by western blot. (E and I) Serum-starved A549 and PC9 cells were stimulated by 50nmol/L PMA for 0, 5, 10, 30, 60min to detect p-PKD2 by western blot. (D, F, H, J) Relative expression of p-PKD2 normalized by GAPDH measured by Image J software.

Full length gels and blots

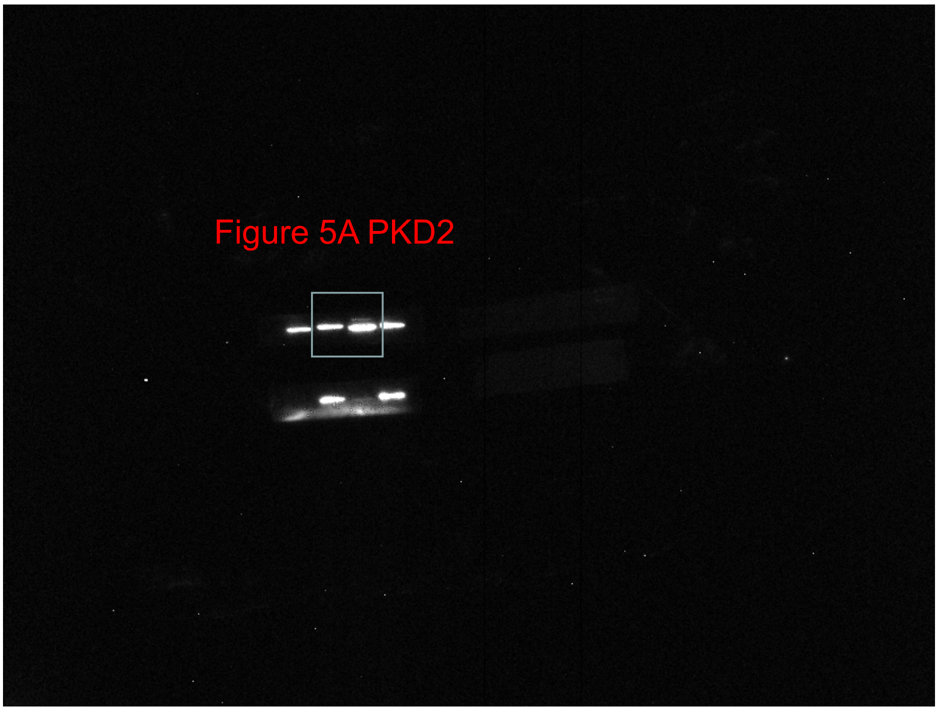

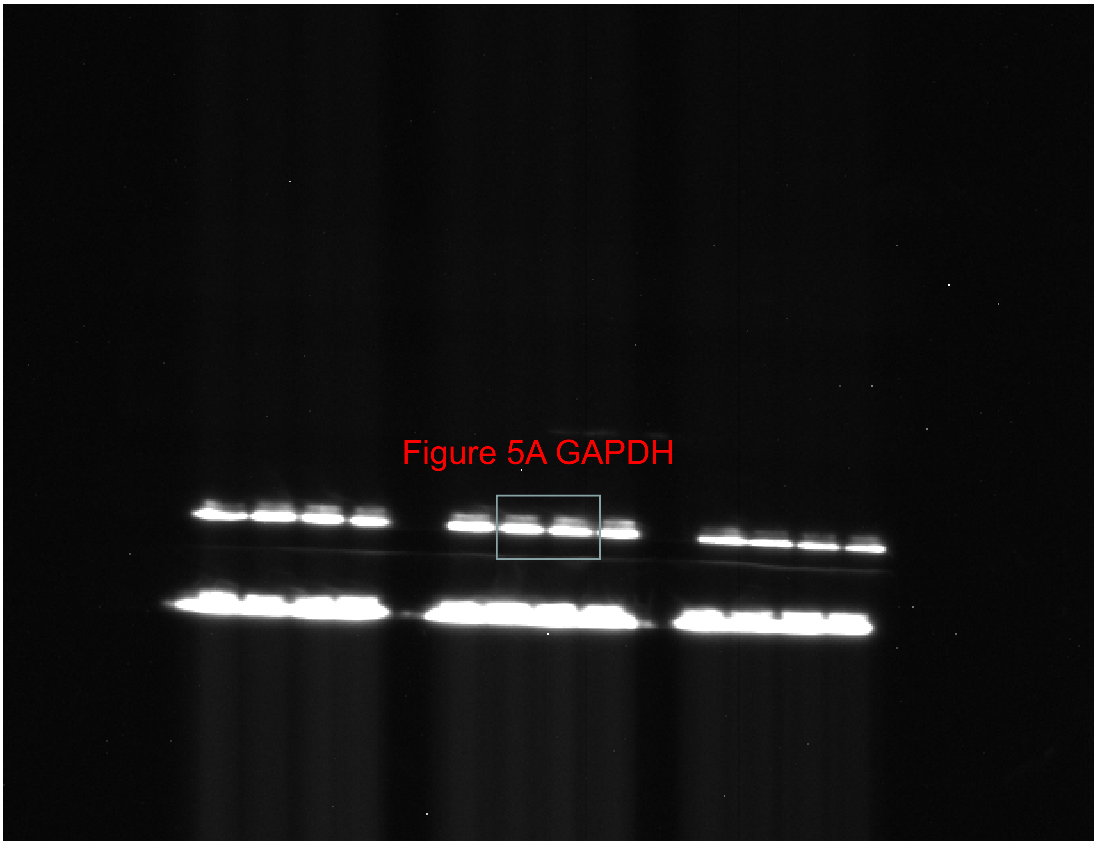
Figure 5A GAPDH

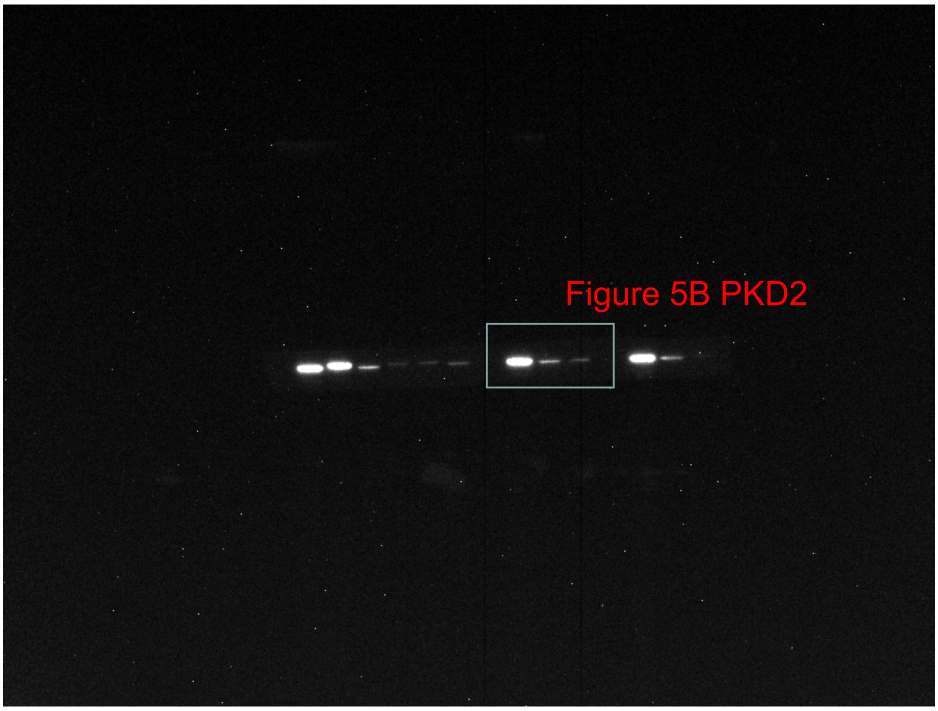
Figure 5B PKD2

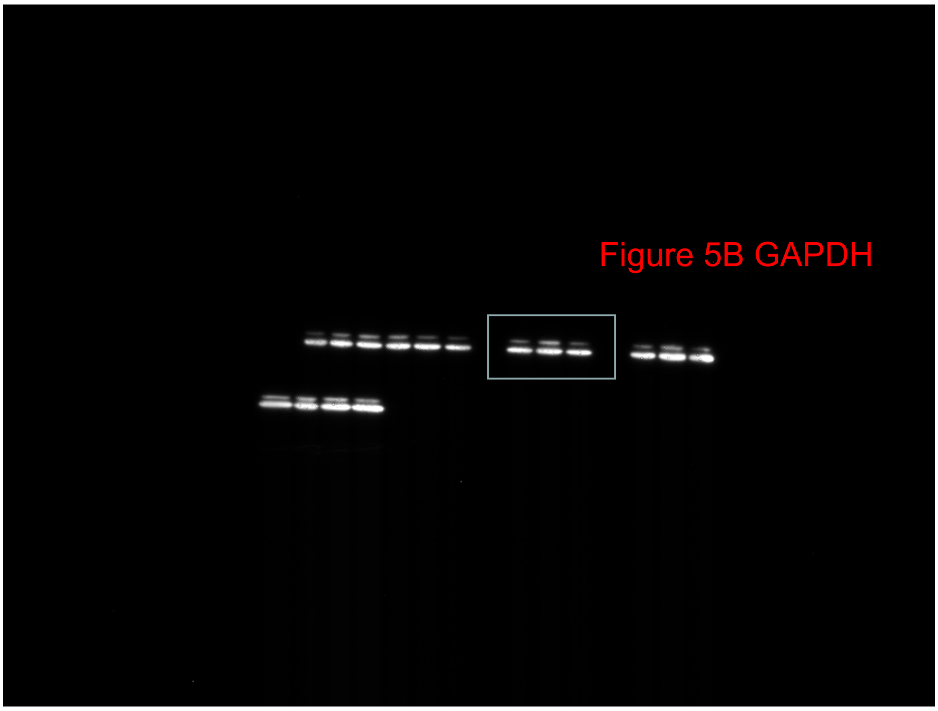
Figure 5B GAPDH

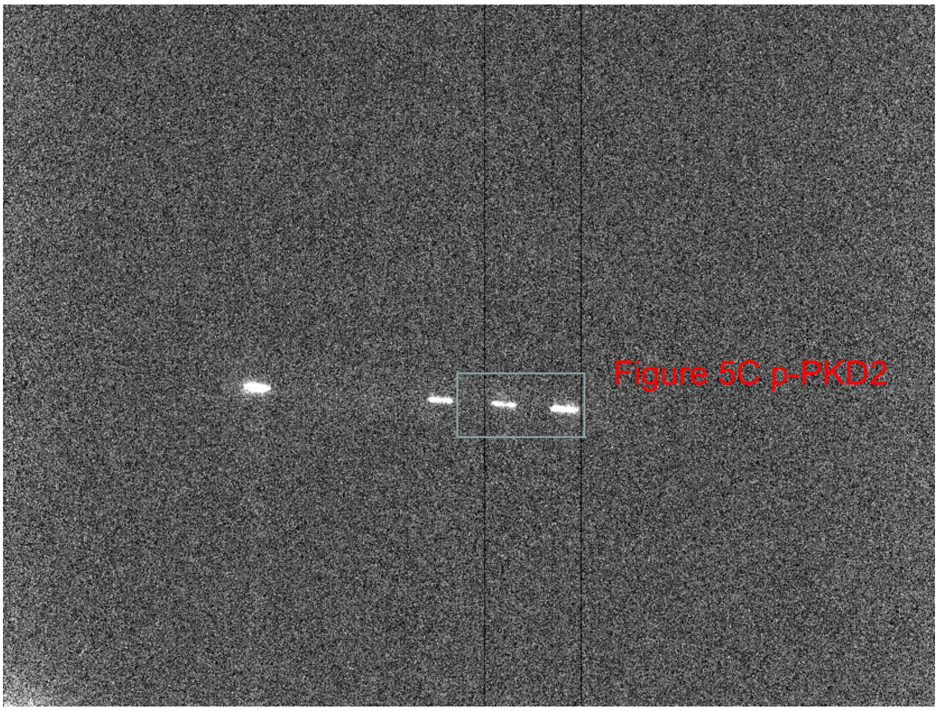
Figure 5C p-PKD2

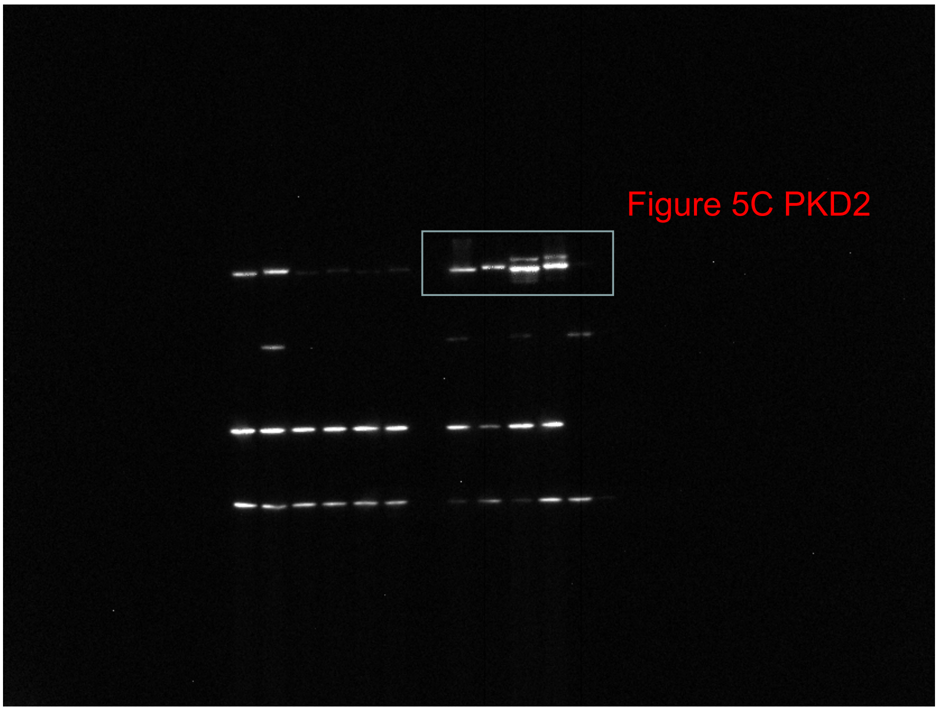
Figure 5C PKD2

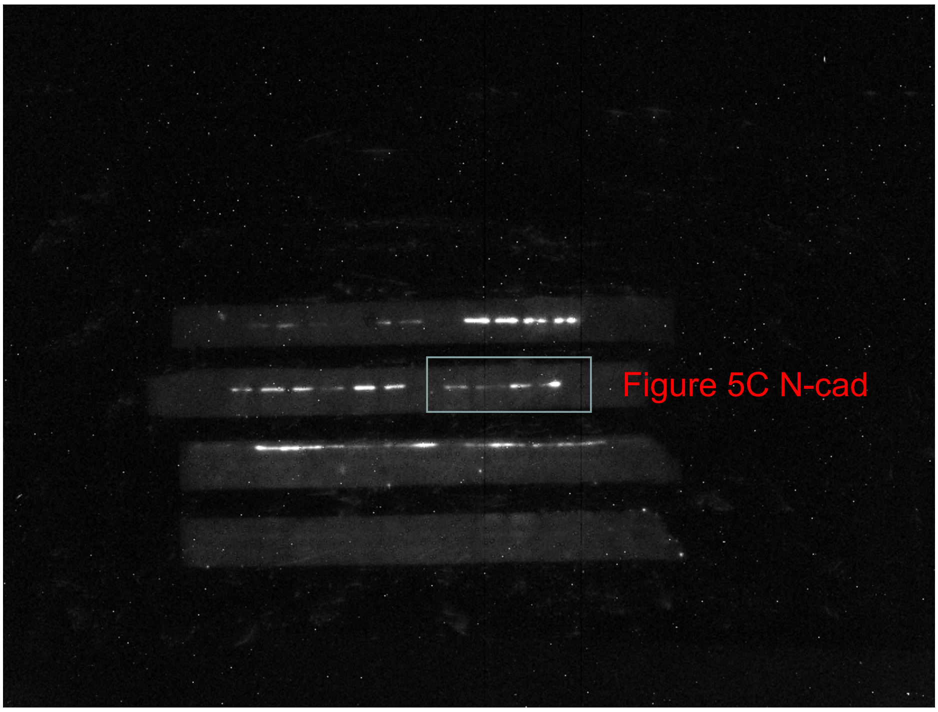
Figure 5C N-cad

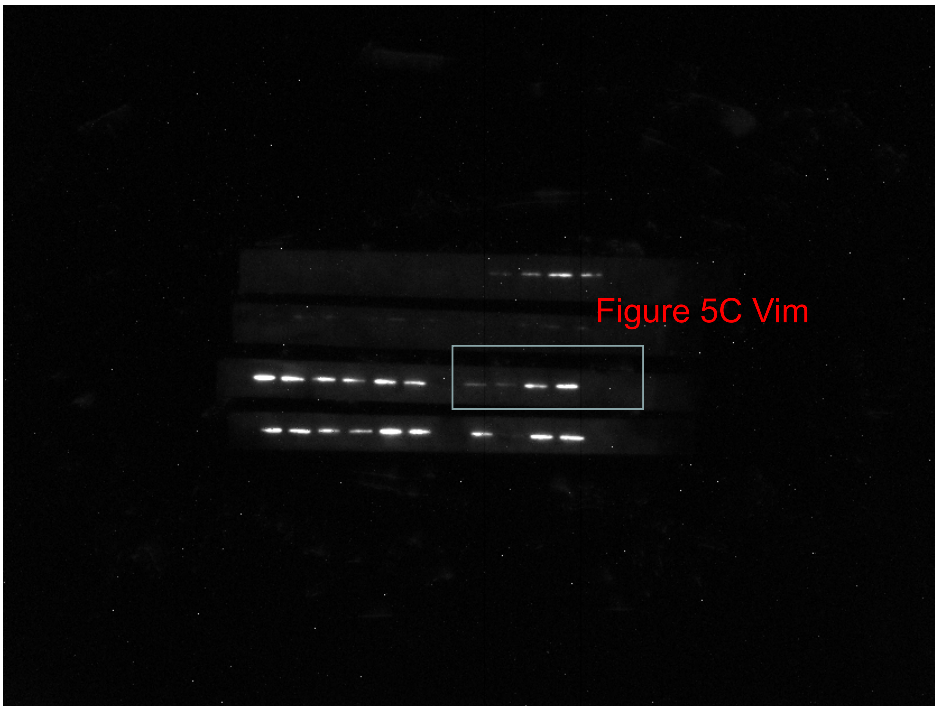
Figure 5C Vim

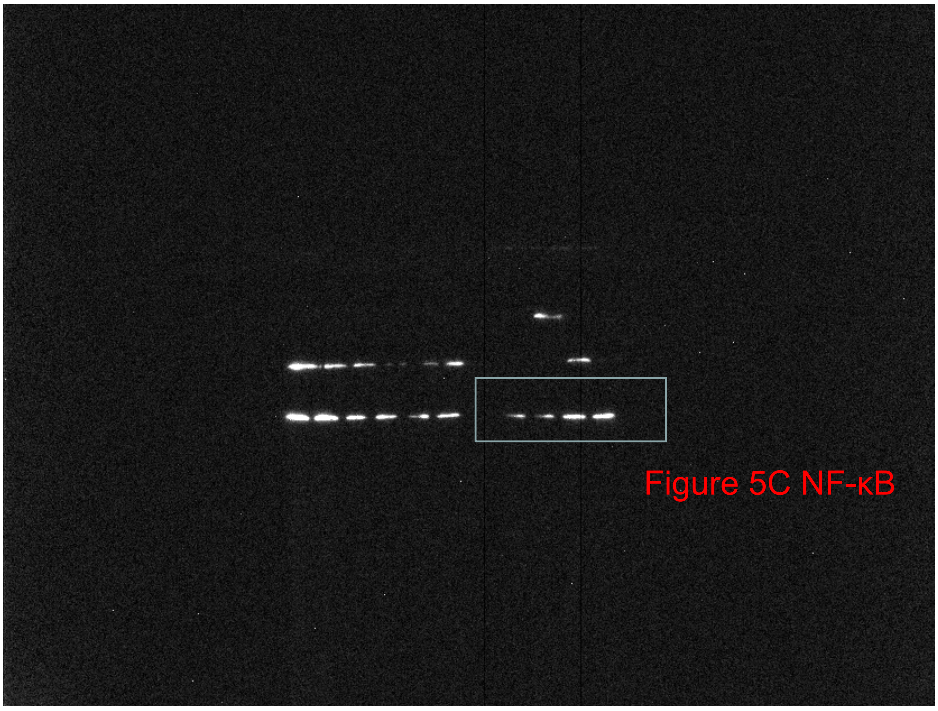
Figure 5C NF-κB

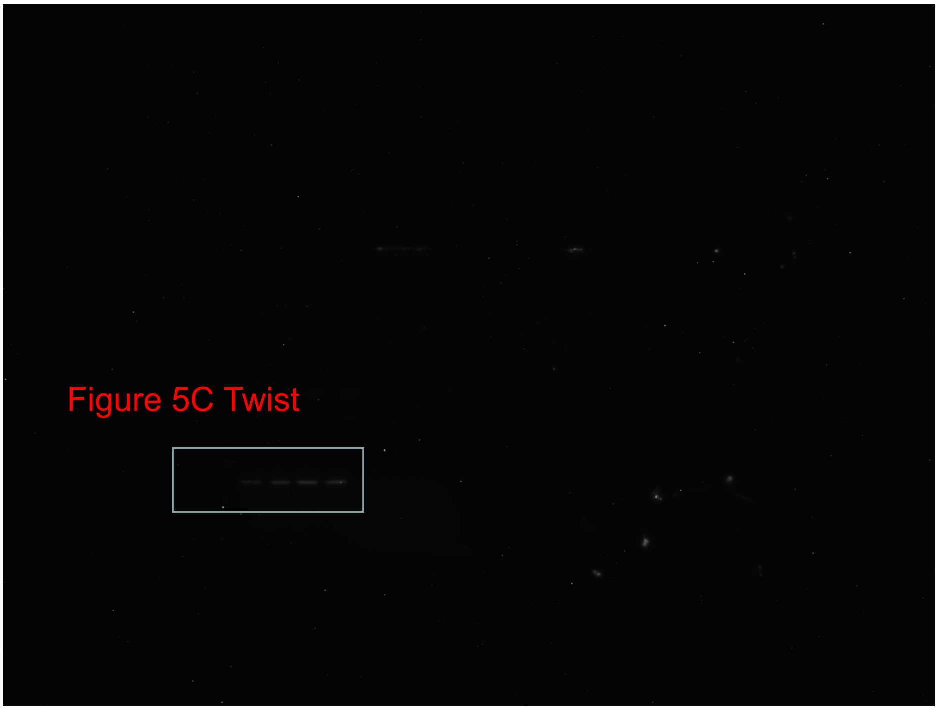
Figure 5C Twist

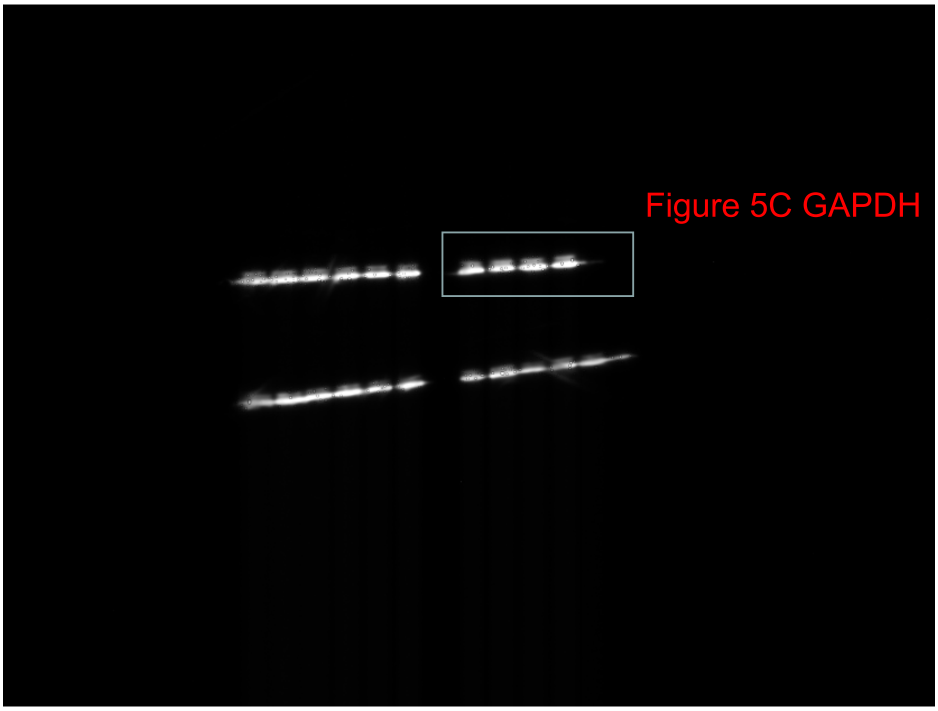
Figure 5C GAPDH

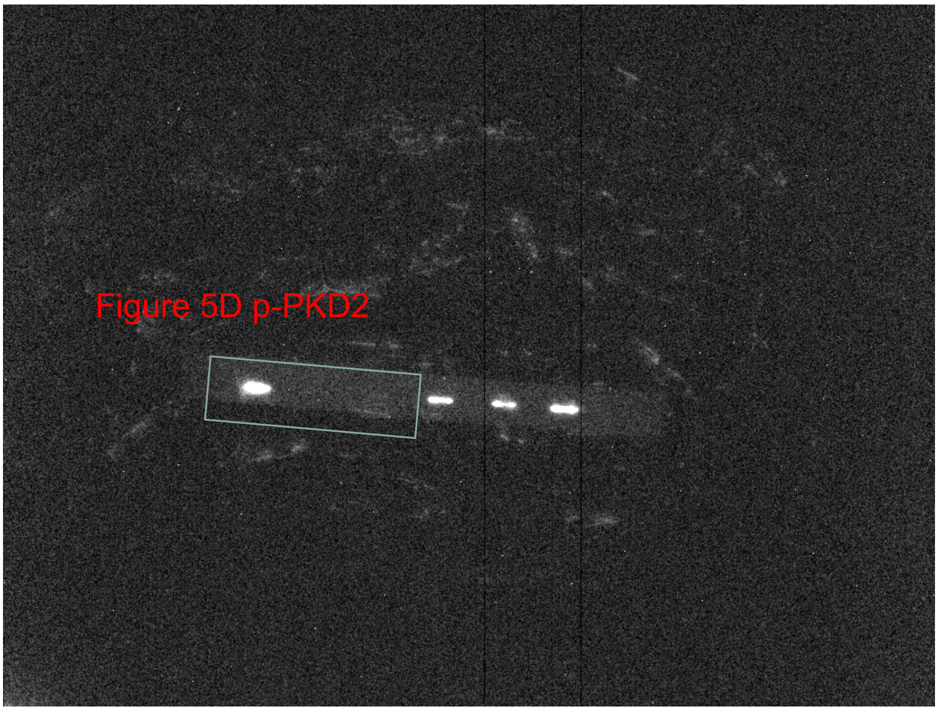
Figure 5D p-PKD2

Figure 5D PKD2

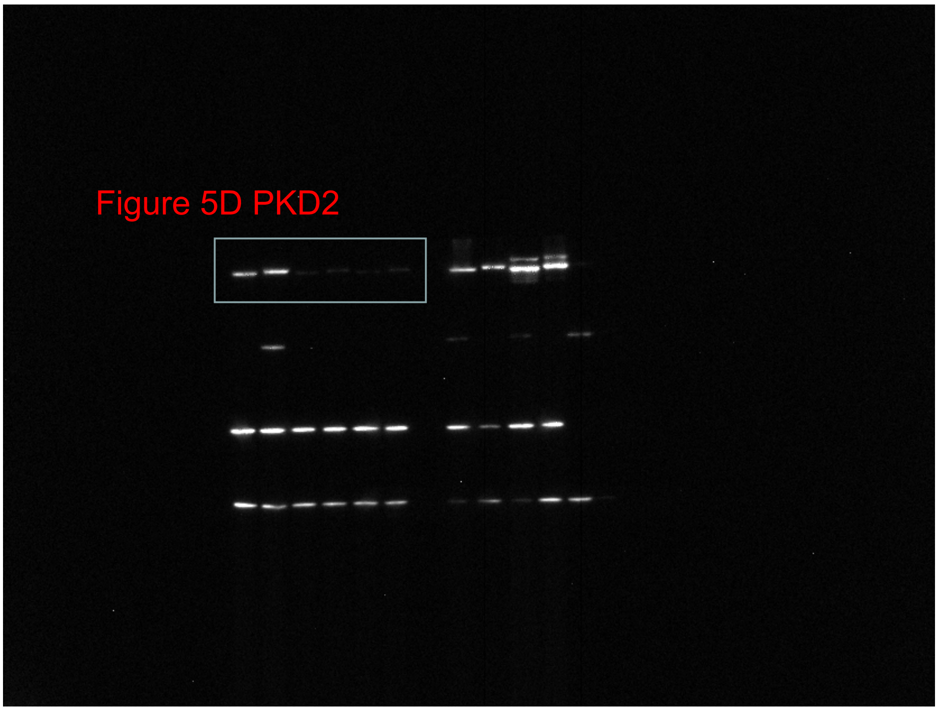

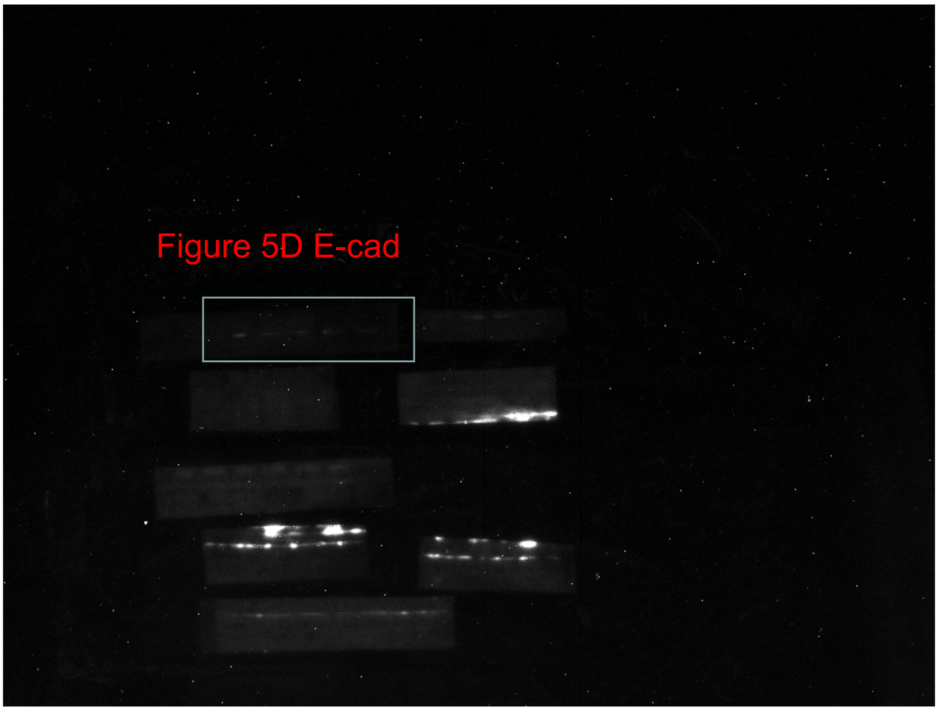
Figure 5D E-cad

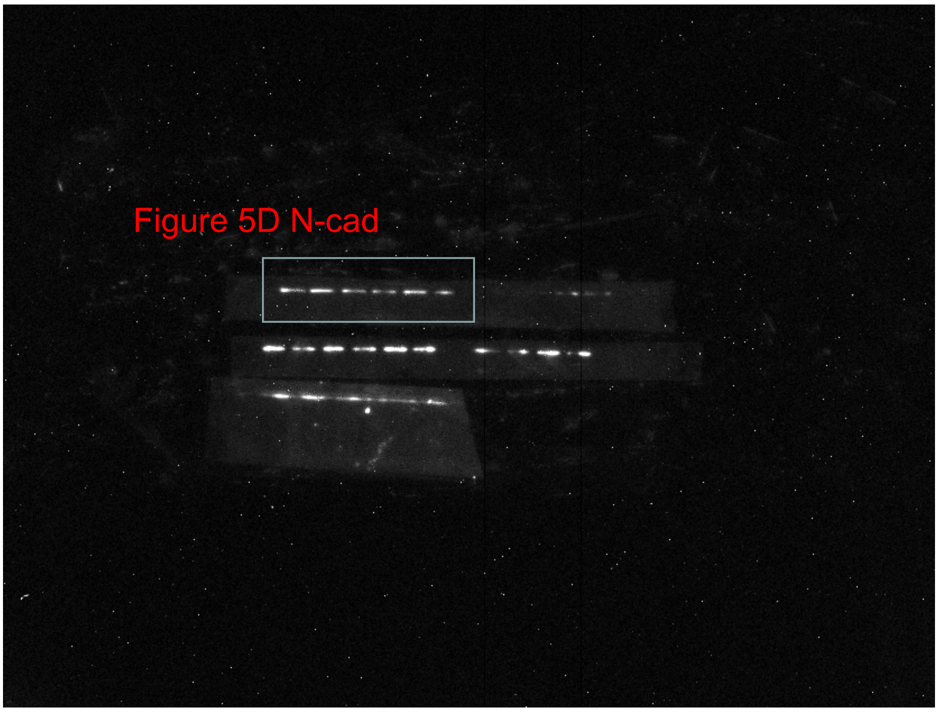
Figure 5D N-cad

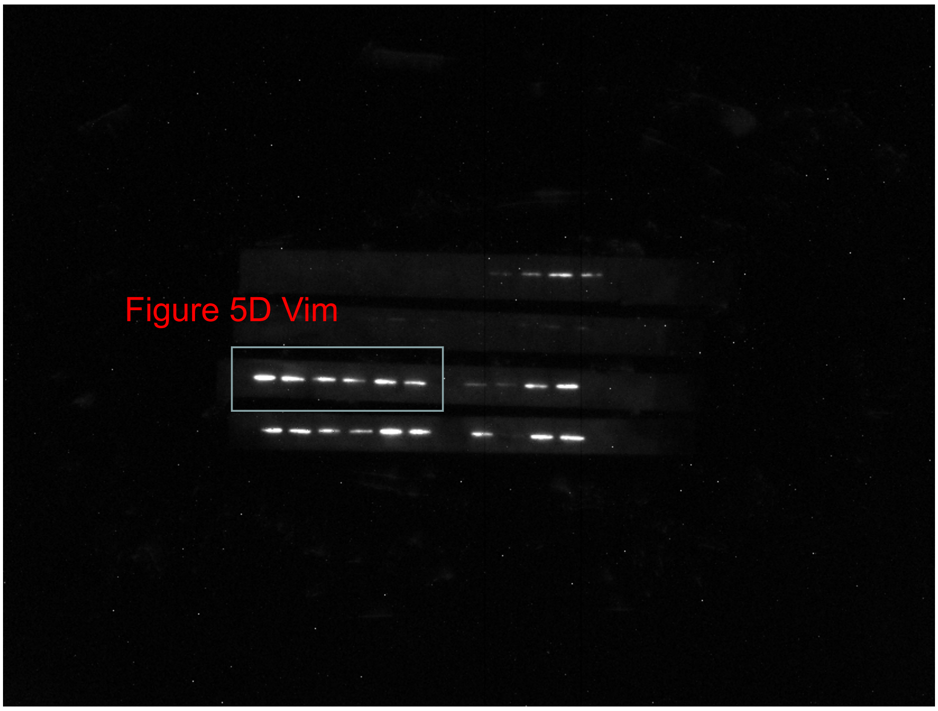
Figure 5D Vim

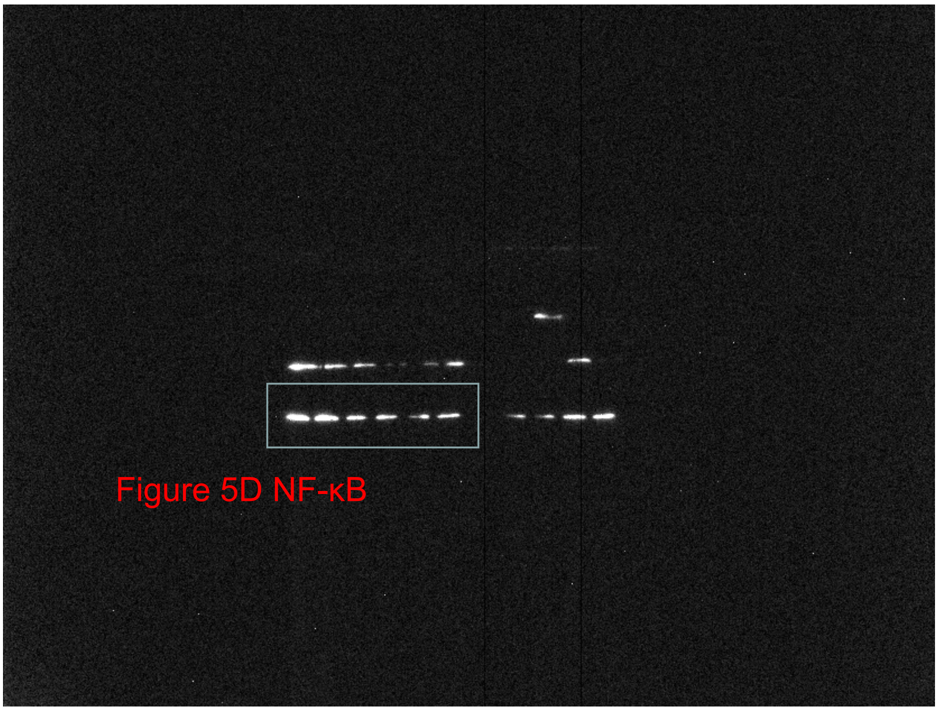
Figure 5D NF-κB

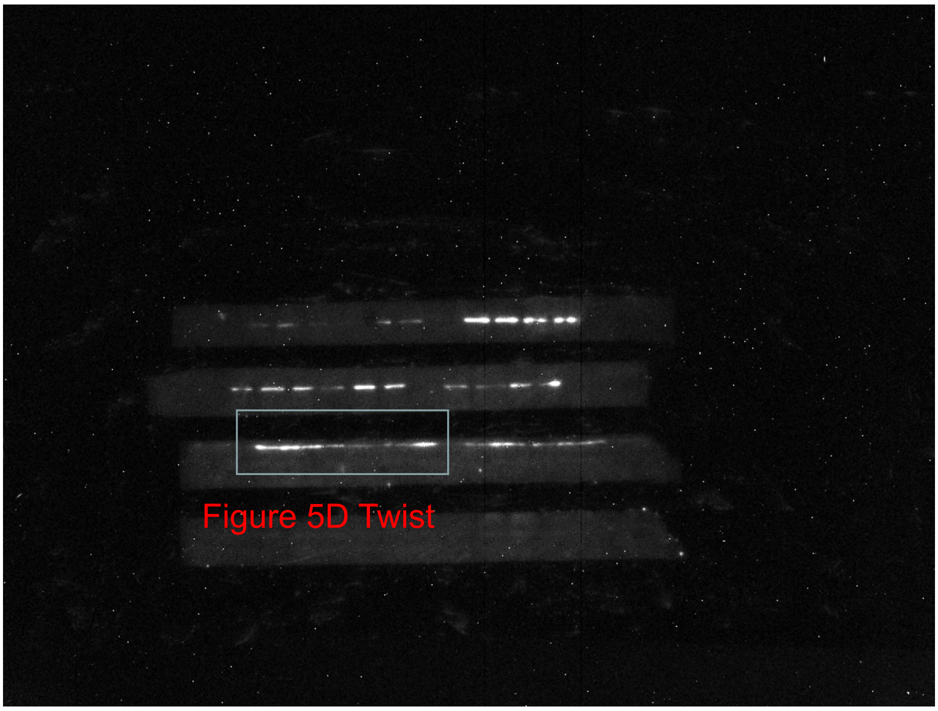
Figure 5D Twist

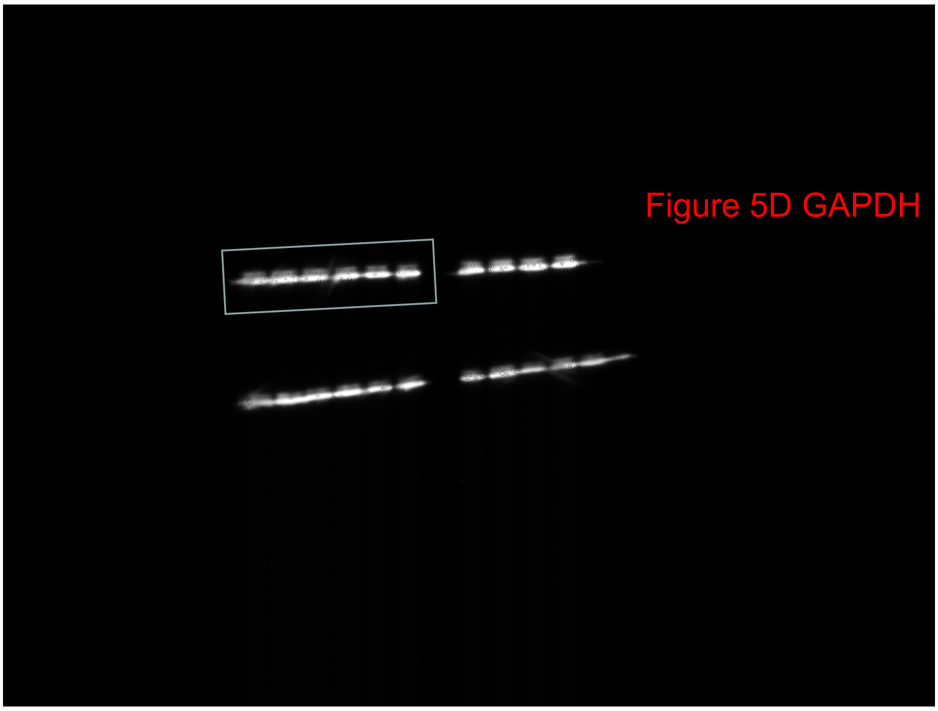
Figure 5D GAPDH

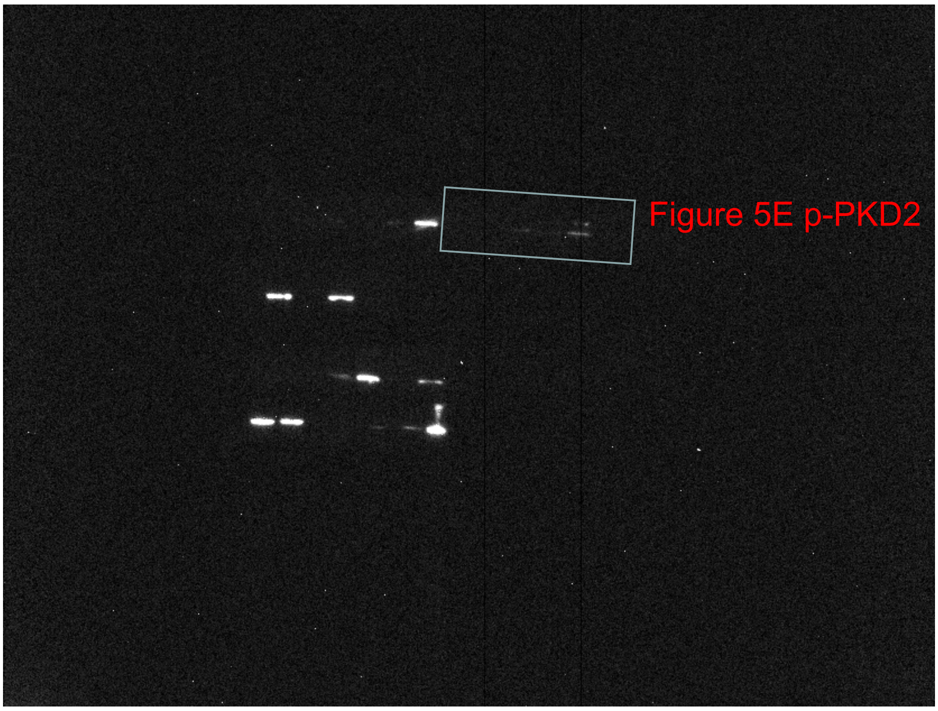
Figure 5E p-PKD2

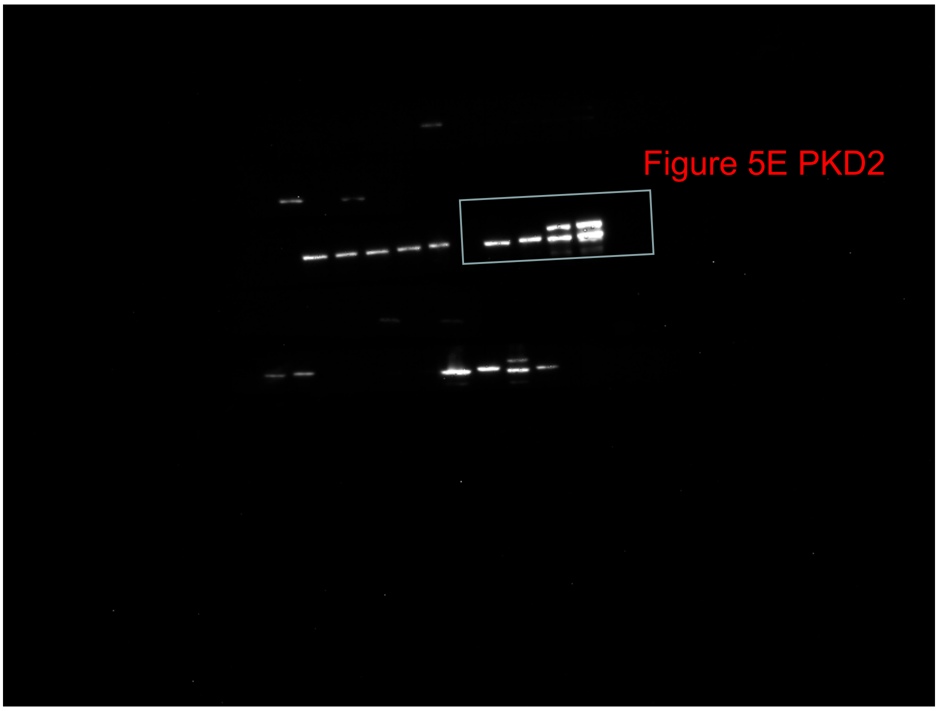
Figure 5E PKD2

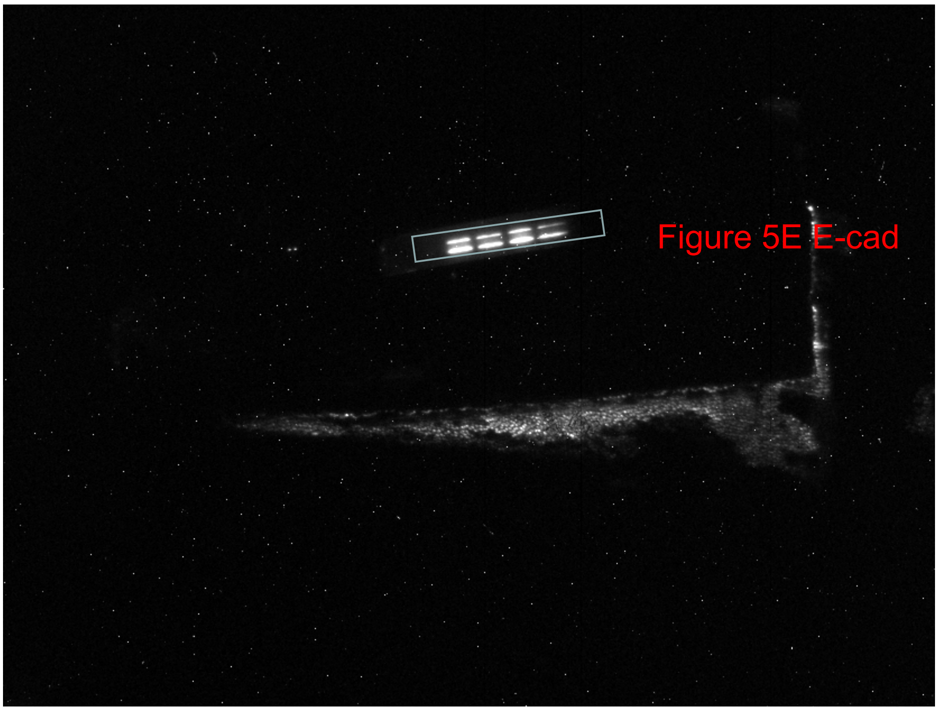
Figure 5E E-cad

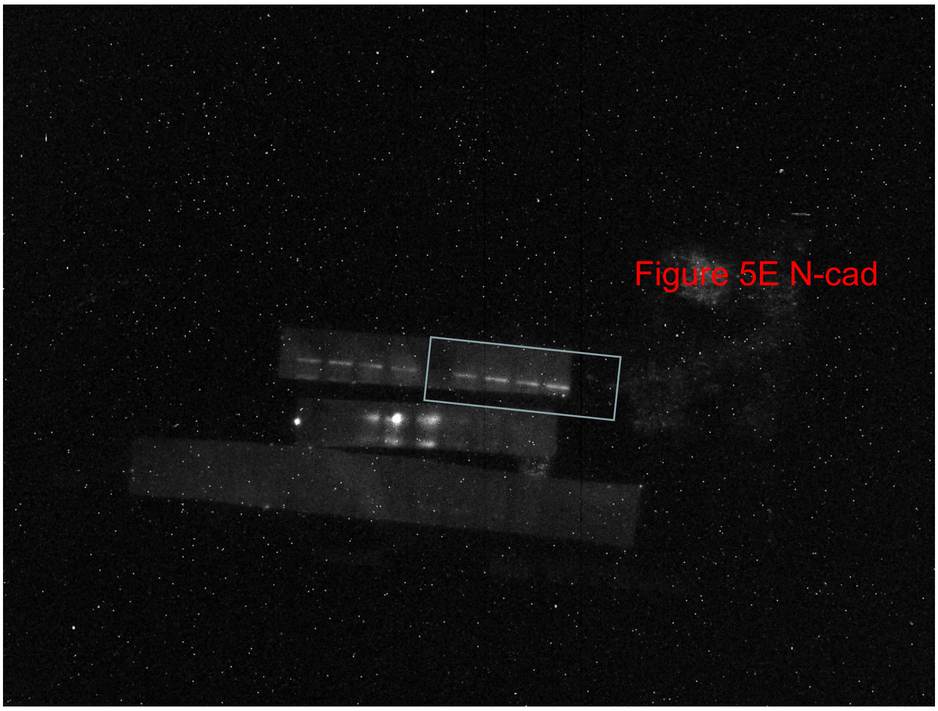
Figure 5E N-cad

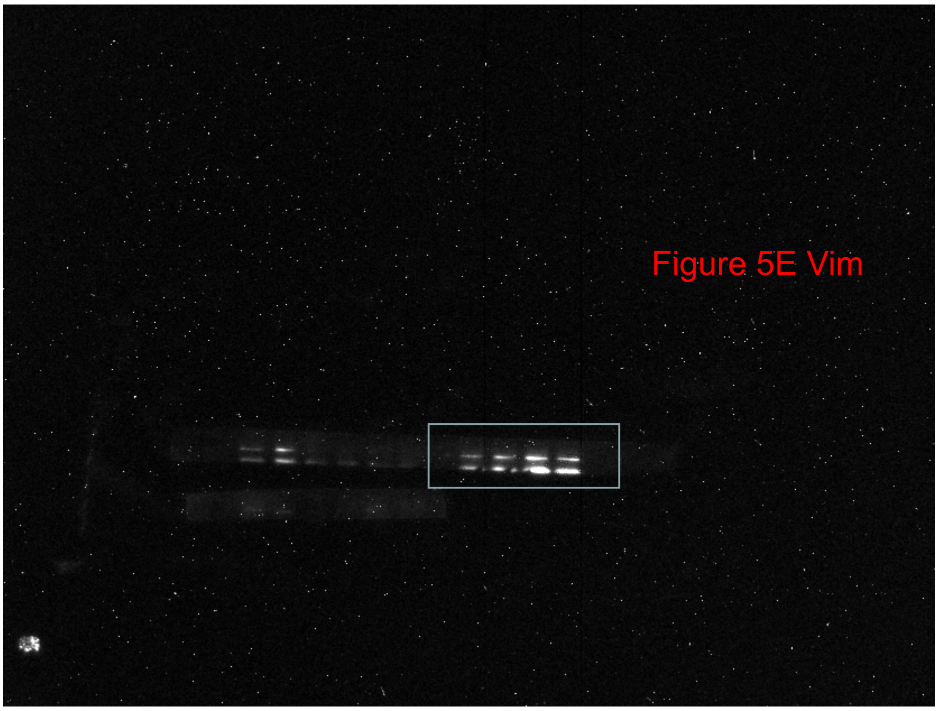
Figure 5E Vim

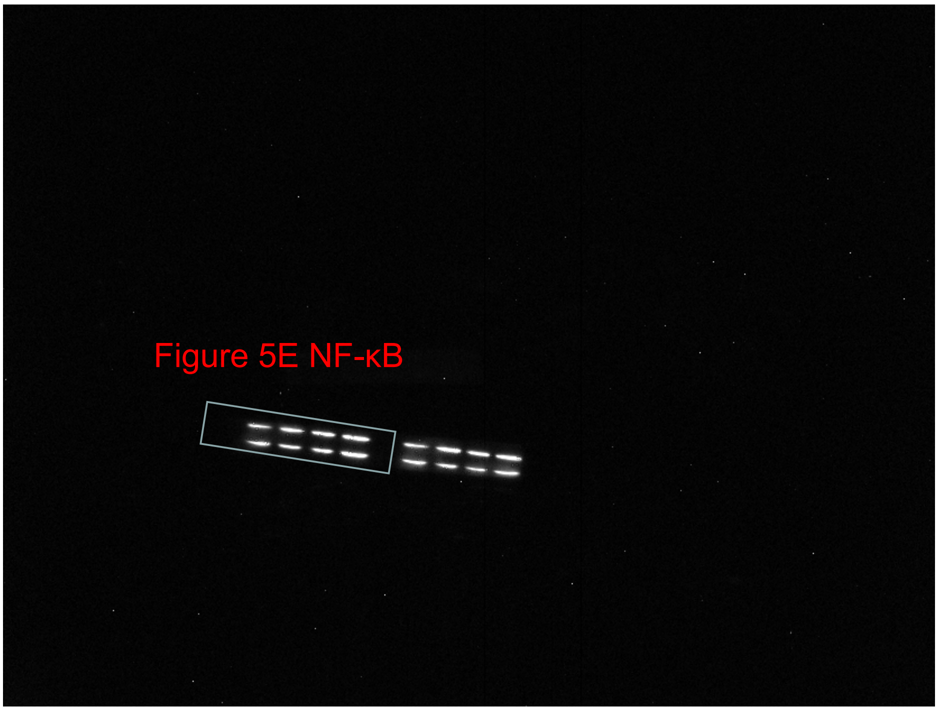
Figure 5E NF-κB

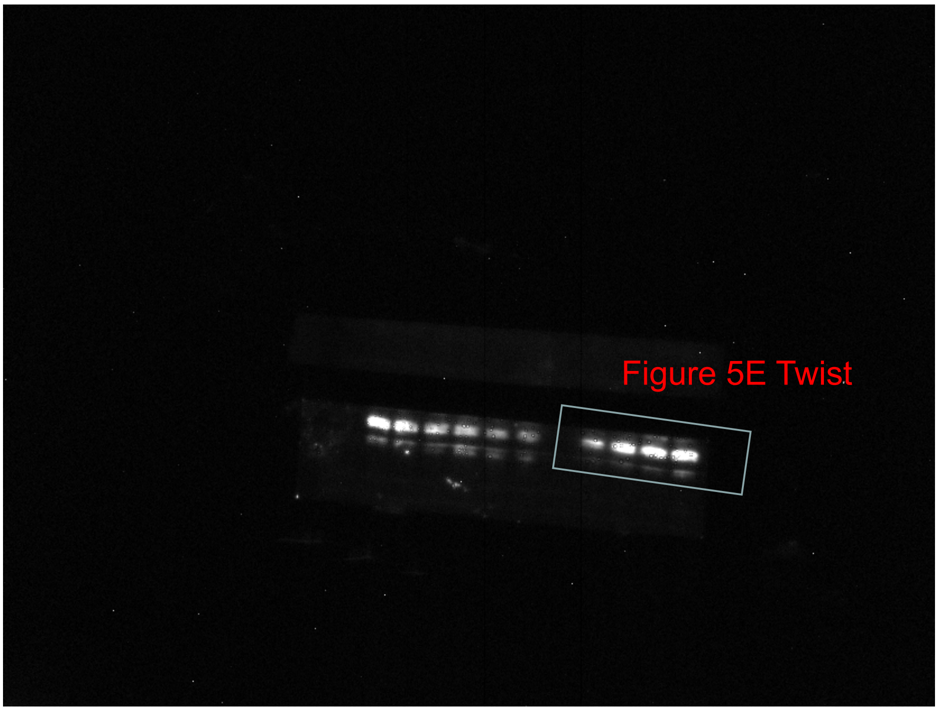
Figure 5E Twist

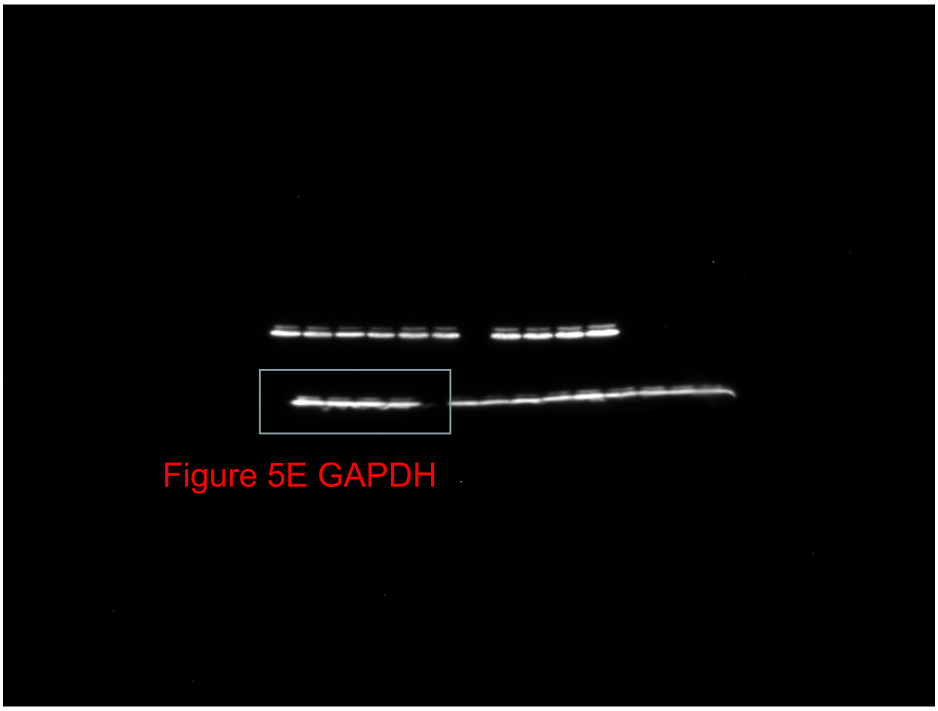
Figure 5E GAPDH

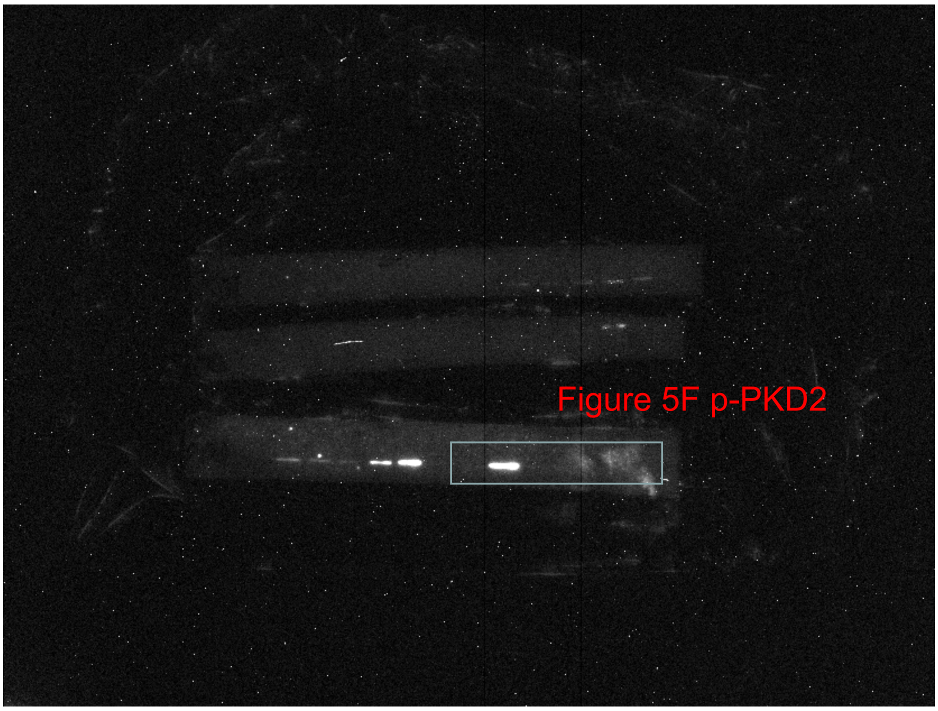
Figure 5F p-PKD2

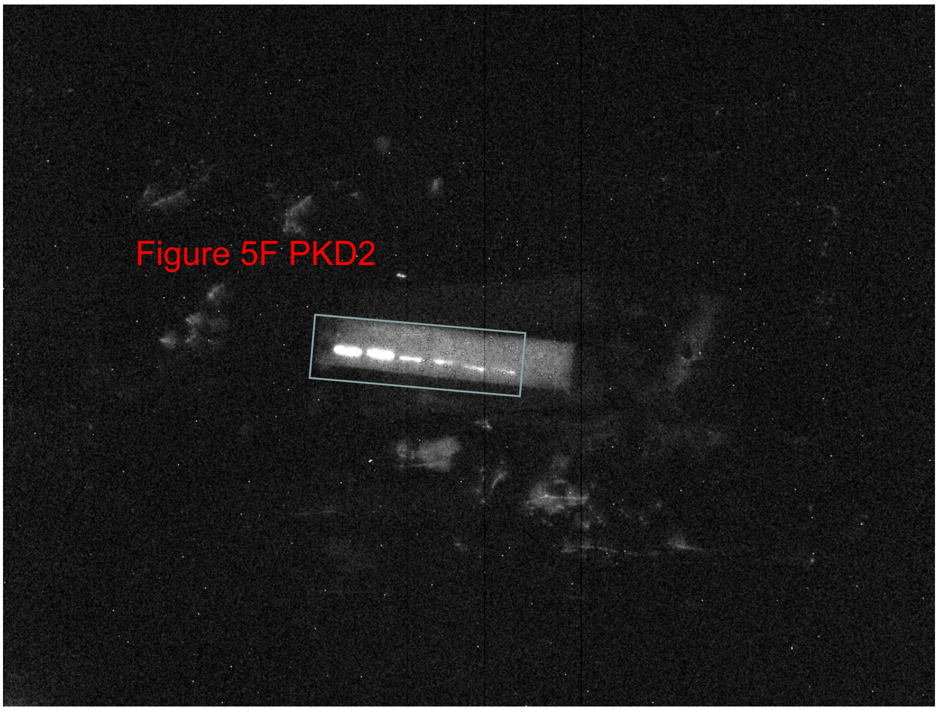
Figure 5F PKD2

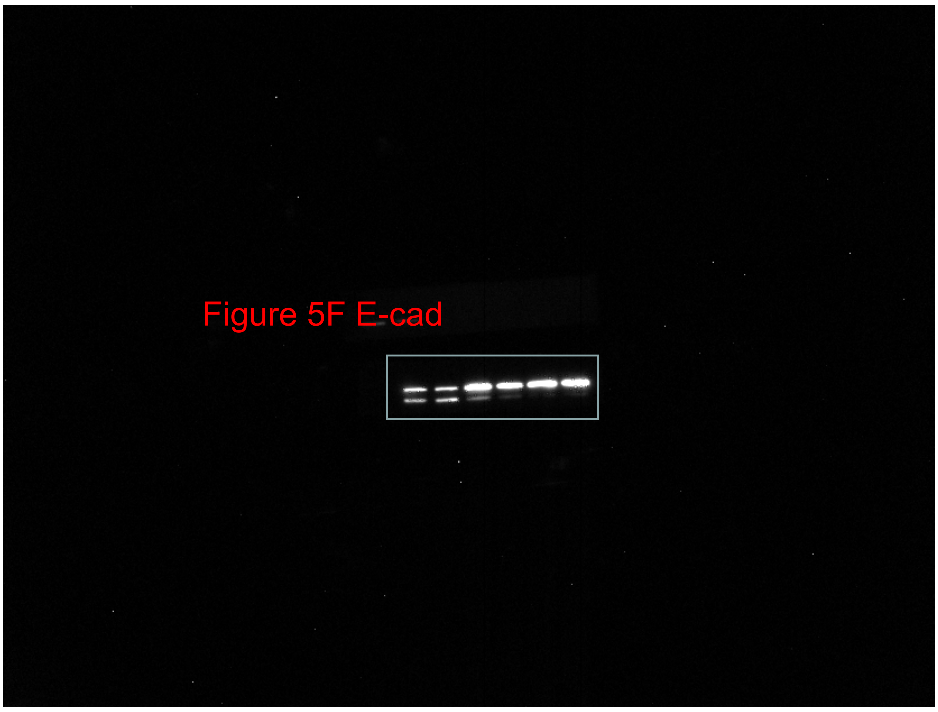
Figure 5F E-cad

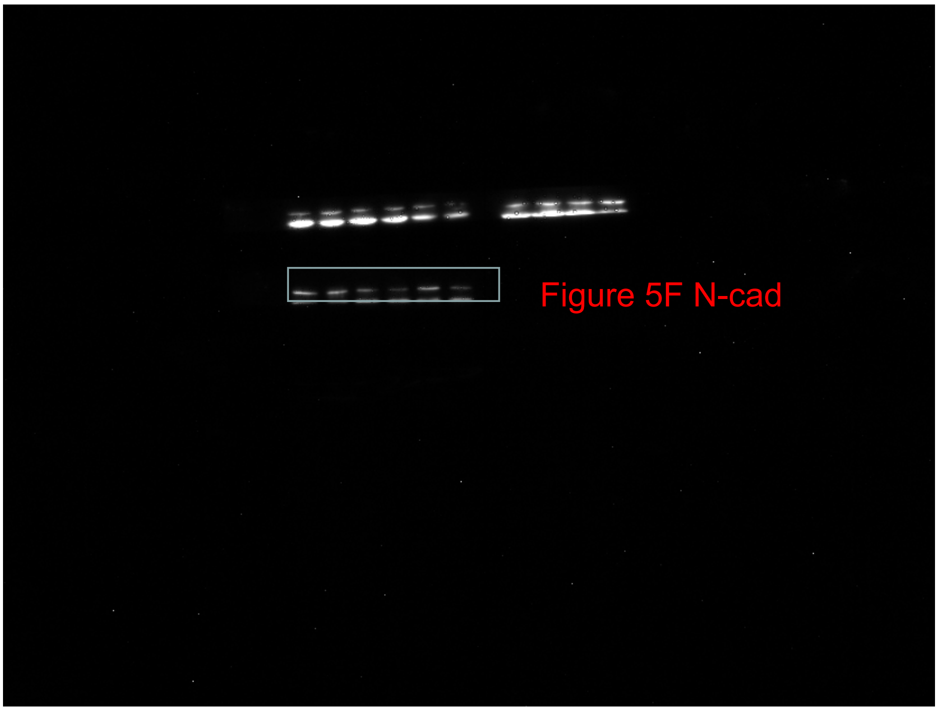
Figure 5F N-cad

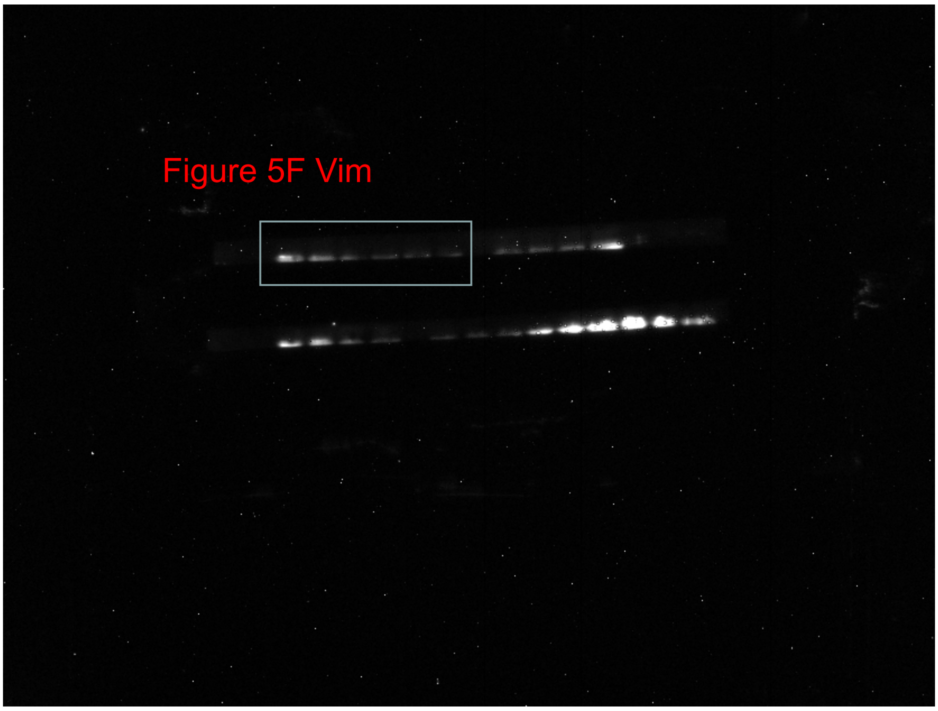
Figure 5F Vim

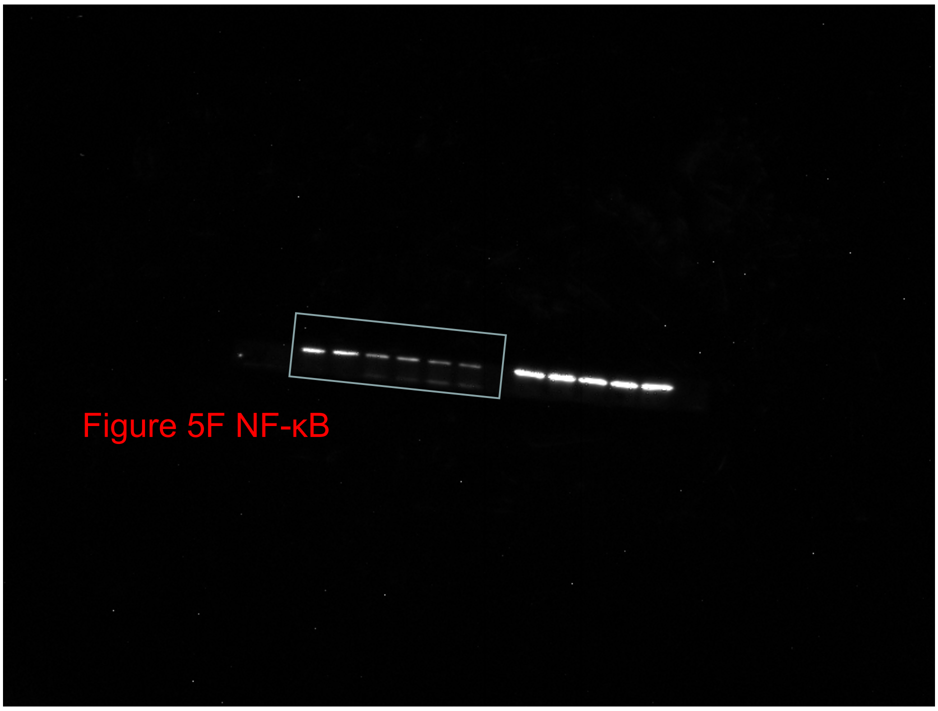
Figure 5F NF-κB

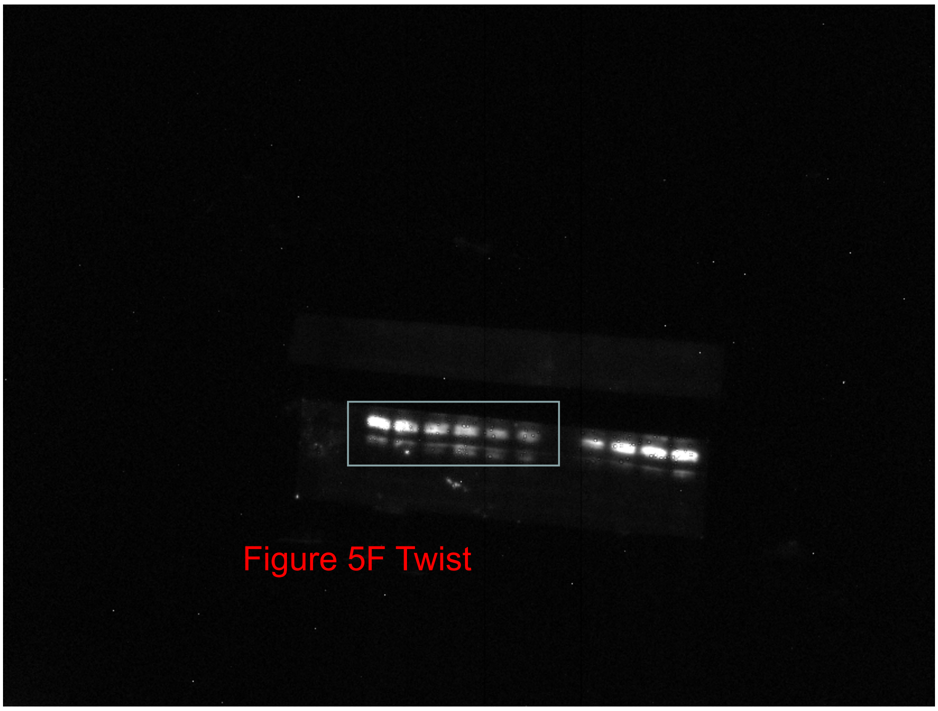

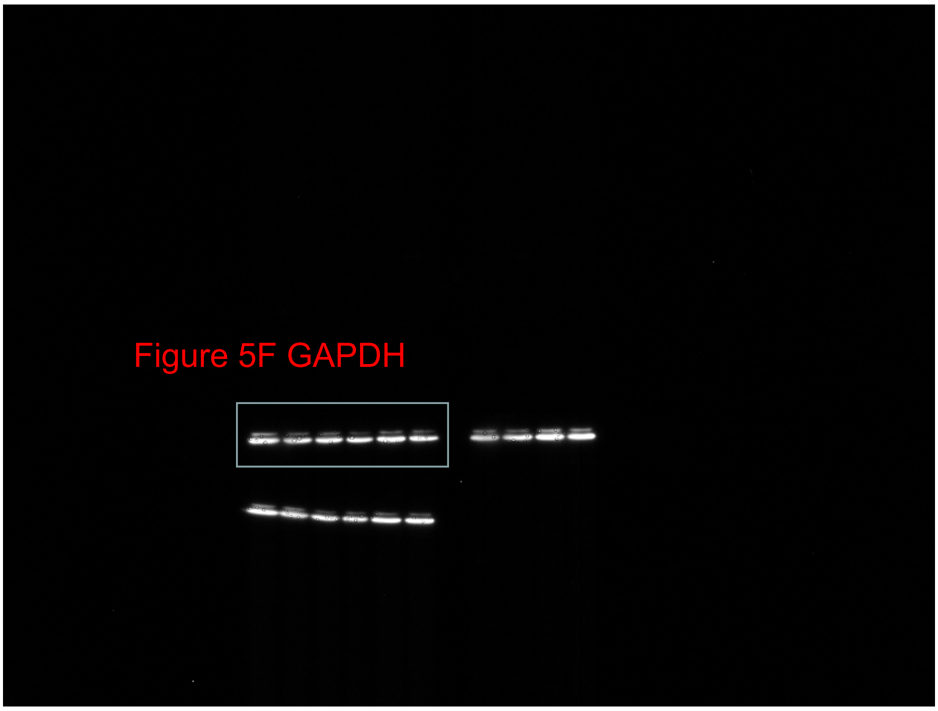
Figure 5F GAPDH
